# Dynamic multi-period optimal power flow considering renewable energy degradation and temperature derating

**DOI:** 10.1038/s41598-026-59492-w

**Published:** 2026-06-29

**Authors:** Bassem Khaled, Almoataz Y. Abdelaziz, Mahmoud A. Attia, Amr K. Khamees

**Affiliations:** 1 Department of Electrical Power and Machines, Faculty of Engineering, Ain Shams University, Cairo, Egypt; 2 Faculty of Engineering and Technology, Future University in Egypt, Cairo, 11835 Egypt; 3 Department of Engineering Physical and Mathematics, Faculty of Engineering, Ain Shams University, Cairo, Egypt

**Keywords:** Climate change, Carbon emissions, Mayfly algorithm, Stochastic optimal power flow, Multi-period optimal power flow, Temperature de-rating, Degradation, Climate sciences, Energy science and technology, Engineering, Environmental sciences

## Abstract

Renewable energy sources, such as solar and wind, play vital role in reducing emissions of carbon dioxide and combating climate change. However, their stochastic behavior has an impact on modern electric networks. Hence, Modelling variability and uncertainty in optimal power flow (OPF) concerns is critical for ensuring dependable and environmentally friendly grid operations. This study investigates the impact of climate change on the integration of wind and solar energy into power systems, with a specific focus on how temperature variations affect the performance and optimal dispatch of wind turbines and solar photovoltaic (PV) systems. To minimize total costs and carbon emissions, both single-objective and multi-objective optimization problems are developed, incorporating temperature-dependent de-rating effects on PV modules and wind turbines. Mayfly algorithm (MA) is applied to the IEEE-30 bus system for Optimal Power Flow (OPF), achieving a 0.6% and 0.5% reduction in fuel cost and carbon emissions compared to PSO and further techniques in single-objective OPF. The study extends to Stochastic OPF (SOPF) on a modified IEEE-30 system with two wind farms and one PV plant. Where, temperature-dependent models are used for both wind energy and solar energy. To additionally encourage and increase the reliance on renewable energy, Carbon credit concept is added to the total cost objective function as novel contribution. The results show that when the carbon credit is taken into account the total cost is reduced by 0.8% compared to the case when it is not considered. To assess the climate change impact, it is found that at 40 °C, the total cost and emissions increase by 21% and 45.67% respectively when minimizing cost, and by 9.67% and 45.7% when minimizing emissions. The multi-period analysis evaluates the evolution of renewable penetration over the 25-year lifetime by considering the gradual reduction in renewable output caused by annual degradation and temperature-related derating. The renewable penetration limits are therefore updated across the planning period to reflect the reduced contribution of PV and wind generation over time. where, this impact is on total cost and carbon emissions over 25-year lifetime through both single and multi-objective dynamic Multi-Period SOPF (MPOPF) problem. For single-objective MPSOPF problem, after 25 years, the results at 40 °C show that the total cost and emissions increase by 24.96% and 51.8% respectively when minimizing the total cost compared to the results obtained at the beginning of wind and solar plants operation. Moreover, Multi-Objective SOPF is solved, a fuzzy-based Pareto front is used, the outcomes show that when the ambient temperature rises to 40 °C, the compromise solution shows an increase of 16.65% total cost and 41.87% carbon emission respectively. For Multi-Objective MPSOPF problem, at 40 °C, the compromise solution shows a 20.16% cost and 60.1% emission increase after 25 years compared to the case when the temperature rise and degradation effect are not taken into account. The findings reveal that climate change and degradation adversely affect renewable energy integration, resulting in increased reliance on thermal power and emphasizing the need for informed planning of sustainable energy infrastructures and re-powering the renewable energy resources at the end of their lifetime.

## Introduction

### Paper motivation

The rising threat of global warming and environmental difficulties is mostly caused by the extensive usage of fossil fuels in electricity generation. As a result, fast advances in renewable energy such as wind and solar farms have rendered them both cost-effective and environmentally benign options. Integrating these sources into contemporary power networks is vital, especially considering their stochastic character^1–3^. However, the key challenge is forecasting, predicting and modeling renewable energy resources^4^. Over the last few decades, wind energy has grown significantly and been widely used. Accurate forecasting is critical since wind speed, the fundamental component in wind energy modelling, fluctuates with time, location, and weather^5^. While for PV generation, solar irradiance is key component in solar energy modeling which is also location and weather dependent^6^.

The Optimal Power Flow (OPF) problem is a complex, nonlinear, and non-convex optimization task aimed at determining the optimal set of control variables to minimize or maximize a given objective function, including fuel costs, carbon emissions, transmission losses, and voltage profiles. while satisfying all operational and physical constraints of the power system^7^. Due to non-convexity, conventional techniques like interior-point approaches and linear and nonlinear programming frequently become stuck in local minima^8–10^. Various metaheuristic algorithms that offer more reliable and effective solution techniques have been developed to address these problems^11–13^. Stochastic renewable resources are now included in recent OPF problems, therefore it’s crucial to simulate both their environmental effects and variability in order to determine how effective they are in reducing emissions and mitigating climate change.

### Literature review

Forecasting wind speed and solar irradiance is necessary to calculate wind energy and photovoltaic power output. Different Probability Distribution Functions (PDFs) such as Weibull distribution, Gamma distribution, Lognormal distribution and other PDF types are among the techniques that the authors employed for wind speed and solar irradiances modeling^14,15^. Several numerical methods have been employed to estimate the parameters of the different distribution functions for wind speed and solar irradiance modeling however; they showed lower accuracy in estimating the distribution parameters^16^. Recently, Artificial Intelligence (AI) optimization algorithms are being used over numerical methods to estimate PDFs parameters due to their higher accuracy. Genetic algorithm (GA), Bacterial Foraging Optimization algorithm (BFOA), Simulated Annealing (SA), Whale Optimization algorithm (WOA) were used to obtain parameters for Weibull, Gamma and Rayleigh PDFs parameters showing better results over classical methods^17,18^. In order to improve model wind speeds, Aquila Optimizer (AO) is used to evaluate two and three component mixture PDFs were implemented in^19^ using Weibull, Gamma, and Inverse Gaussian PDFs where, The outcomes demonstrated that mixed PDFs were able to deliver a more accurate results. In^20,21^, Lognormal PDF was used to model the stochastic behavior of solar irradiance showing better representation over various PDFs. Three component mixture distribution was used in^22^ by combining the Weibull and Gamma PDFs to model solar irradiance where the results demonstrated a reduction in the error between real data and the distribution curve. Both traditional and stochastic OPF problems have been successfully solved using a variety of metaheuristic optimization techniques. Harris Hawk Optimization (HHO) outperformed Butterfly (BF), Ant Lion Optimizer (ALO) were used in^23^ to solve SOPF problem to minimize the total fuel cost and power losses. While in^12,24^, Fuel cost, power loss, and Voltage Stability Index (VSI) were reduced by using the Marine Predator Algorithm (MPA) and Self-Learning JAYA to solve single- and multi-objective OPF problems. In addition, total power losses are minimizd to optimally size and locate Distributed Generation in an Unbalanced Distribution System of IEEE 34 and 123 bus systems moreover, particle swarm optimization (PSO) is used to for optmal sizing and placement of Distributed Generation including UPQC^25,26^. The Enhanced Walrus Optimization (EWO) and weighted mean of vectors INFO methods were used to reduce overall cost and cut down on convergence time including wind and solar systems^27,28^. Developed PSO over-performed the classic PSO in^29^ when it was used to solve SOPF problem in IEEE 30 bus system to reduce overall fuel cost, power losses, and exhaust. Improved Pelican Optimization Algorithm (IPOA) to solve a stochastic optimal power flow problem by integration of solar and wind energy to attain the lessen generation cost without and with inclusion of emissions where, the results were enhanced compared various algorithms^30^. Modified Cheetah Optimizer (MCO) was applied in^31^ to solve single-objective SOPF problem to minimize overall operating cost, voltage deviation, pollutant emissions, and power loss. Artificial Gorilla Troops Optimization was used in^32^ to solve the SOPF problem in the hybrid grid considering wind and solar uncertainties. The White Shark Optimizer (WSO) was assessed for solving the OPF problem with wind energy penetration focusing on minimizing the overall system cost^33^. Boosting Circulatory System Based Optimization (BCSBO) was used to solve SOPF problems with wind and solar penetration to minimize total cost including carbon tax and voltage deviation. The results showed significant reduction compared to classical algorithms^34^. Elephant Herd Optimization (EHO) was used to solve SOPF problem with the objective to minimize generation costs, emission, voltage stability, and losses^35^.

In^36^, the Adaptive Lightning Attachment Procedure Optimizer (ALAPO) was employed to reduce voltage variation and power loss. In order to solve both single-objective and multi-objective SOPF issues, the Modified Rao-2 (MRao-2) algorithm and HOA were evaluated. They improved global search capabilities while minimising overall cost, power loss, and voltage variations^37,38^. In a test system using renewable energy resources, the hybrid crow search algorithm (CSA) and JAYA were utilised to give the best scheduling in combined economic emission dispatch, resulting in better and higher quality solutions^39^. The MA was applied to solve both single-objective and multi-objective SOPF problems, considering mixed distribution functions for renewable resources^40^. Single-objective and multi-objective SOPF problems were addressed using the Tasmanian Devil Optimization (TDO) algorithm with the objective to minimize the total generation cost^41^. In^42^, the Hybrid Firefly–JAYA Algorithm was applied to multi-objective SOPF issues with both solar and wind resources present. Furthermore, the SOPF problem was solved with improved exploitation capabilities by a Hybrid Jellyfish Search (JS) and Moth Flame Optimizer (MFO) combination, which aimed to minimise fuel expenses and power losses^43^. The ambient temperature effect on wind energy was assessed in^44^, where, single and multi-objective SOPF were solved using Exponential Distribution Optimizer (EDO). The results showed the impact of temperature rise on the total generation cost and carbon emissions.

A number of difficulties in addressing the SOPF problem are apparent from the examined studies, and these might be summed up as follows:

- There is a lack in correlation between actual PV and wind turbine performance and the theoretical models commonly employed in wind energy modelling.
- The failure to account for how climate change may affect the uptake of renewable energy, which has a big impact on fuel prices and carbon emissions.
- The promotion for Carbon Credits as an encouragement for renewable energy penetration isn’t taken into account in SOPF problem.
- The degradation of renewable energy resources is not considered where, the degradation will impact the total operation cost and carbon emissions during the renewable energy projects’ lifetime.

### Paper contribution and organization

This work proposed the following innovative strategies to fill the gaps that have been identified in the literature survey:

- Using temperature-dependent wind and solar energy models to assess SOPF problem at different temperature ranges to determine how climate change affects fuel costs, carbon emissions, and power losses.
- Employing Carbon Credit principle as a novel contribution in solving single and multi-objective SOPF problem to increase the reliance on renewable energy sources.
- Assessing the degradation effect of renewable energy resources on the total operation cost and carbon emissions during the renewable energy projects’ lifetime.
- Applying Mayfly algorithm (MA) to solve both OPF and SOPF issues and determine as well the PDF parameters in order for wind and solar energies to outperform more conventional optimization methods.

This work investigates the Optimal Power Flow (OPF) problem using the IEEE-30 bus test system to evaluate the performance of the MA algorithm. Single- and multi-objective Stochastic OPF (SOPF) problems are formulated on a modified IEEE-30 network with two wind farms and one solar farm, aiming to minimize both operating costs and carbon emissions while incorporating carbon credit incentives to promote renewable integration. A major contribution of this study is the incorporation of temperature-dependent wind and solar models into the SOPF problem. Furthermore, multi-period SOPF problems are solved over a 25-year period, considering wind turbine and PV module degradation. The findings show that renewable aging and temperature variations significantly increase operational costs and emissions, underscoring their importance in long-term energy and climate change mitigation planning. This paper is organized as follows: Sect. “Renewable energy modeling” describes the modeling of renewable energy resources wind and solar. Section “Optimal power flow” presents the problem formulation of single and multi-objective OPF problems. The MA algorithm used to solve the OPF problem is presented in Sect. “Mayfly (MA) algorithm”. Section “Results and discussion” presents the results and a discussion of the findings. Finally, the conclusion is drawn in Sect. “Conclusion”.

## Renewable energy modeling

### Wind speed modeling

The Weibull distribution function is developed by Weibull^45^. It’s a two- parameter PDF where, the wind speed probability distribution function, $f\left(v\right)$ and cumulative distribution function $F\left(v\right)$ are given by Eqs. (1) and (2) respectively.

1 $$f_{W} \left( v \right) = \frac{k}{{c^{k} }}v^{{v - 1}} {\mathrm{exp}}\left( { - \left( {\frac{v}{c}} \right)^{k} } \right)$$

2 $$F_{W} \left( v \right) = 1 - {\mathrm{exp}}\left( { - \left( {\frac{v}{c}} \right)^{k} } \right)$$

where, $v$ represents the wind speed, *k* and *c* are the shape and scale factors, respectively.

### Wind turbine modeling

Environmental conditions play a critical role in the performance of renewable energy resources. For wind turbines, the maximum achievable power output,${P}_{max}$, is directly influenced by ambient temperature,${T}_{a}$. This relationship is captured through active power de-rating curves, which reflect the gradual reduction in output as temperatures exceed manufacturer-defined thresholds. Elevated ambient temperatures lead to internal heat accumulation and decreased cooling efficiency, ultimately necessitating output curtailment. If the temperature reaches a critical limit, the turbine undergoes a protective shutdown to safeguard its electrical components as explained in^46^ using G80 windturbine. where, the temperature-dependent model is deployed in this study. Equations (3), (4) and (5) describe the model.

3 $$P_{{max}} = \left\{ {\begin{array}{*{20}l} {0v < v_{{cut - in}} } \hfill \\ {P_{u} \left( {\frac{{v - v_{{cut - in}} }}{{v_{u} - v_{{cut - in}} }}} \right)v_{{cut - in}} \le v \le v_{{max}} } \hfill \\ {P_{u} v_{u} \le v \le v_{{cut - out}} } \hfill \\ \end{array} } \right.$$

4 $$v_{u} = v_{{cut - in}} + \left( {\frac{{P_{u} }}{{P_{r} }}*\left( {v_{r} - v_{{cut - in}} } \right)} \right)$$

5 $$P_{u} = \left\{ {\begin{array}{*{20}l} {P_{r} T_{a} \le T_{d} } \hfill \\ {P_{r} - d(T_{a} - T_{d} )T_{d} \le T_{a} \le T_{o} } \hfill \\ {0T_{d} > T_{o} } \hfill \\ \end{array} } \right.$$

where, ${P}_{r}$ is the wind turbine rated power without de-rating, ${v}_{cut-in}$ is the cut-in wind speed, $v$ is the wind speed, ${v}_{r}$ is the rated wind speed and ${v}_{cut-out}$ is the cut-out wind speed. ${P}_{u}$ is the ultimate power that could be generated at ${v}_{u}$ when the wind turbine is de-rated. ${T}_{d}$ is the temperature at which the de-rating curve starts. $d$ is the slope of the de-rating curve. ${T}_{o}$ is the maximum temperature at which the turbine can operate.

### Solar irradiance modeling

The lognormal distribution is a special case of a normal distribution with random variable logarithm is normally distributed^47^. The probability distribution of solar irradiance can be stated using the Eq. (6).

6 $${f}_{l}\left(I, \upmu ,\upsigma \right)=\frac{1}{\mathrm{I} \upmu \sqrt{2\pi }} \mathit{exp}\left(-\frac{1}{2}{\left( \frac{\mathrm{ln}I-\upsigma }{ \upmu }\right)}^{2}\right)$$

where, $I$ is solar irradiance, $\upmu$ is the mean and σ is the standard deviation.

### Solar PV modeling

The solar PV output power ${P}_{sm}$ is generally described in Eq. (7)^48^. Ambient temperature plays a critical role in reducing the solar PV output power. Hence, Eqs. (8) and (9) describe the solar PV output power depends on the ambient temperature^49^.

7 $$P_{s} \left( I \right) = \left\{ {\begin{array}{*{20}l} {P_{{sm}} \left( {\frac{{I^{2} }}{{I_{{std}} R_{c} }}} \right),for0 < I < R_{c} } \hfill \\ {P_{{sm}} \left( {\frac{I}{{I_{{std}} }}} \right),forI > R_{c} } \hfill \\ \end{array} } \right.$$

8 $${P}_{sm}= {P}_{r,STC} (1- \upgamma \left({T}_{PV}- 25\right))$$

9 $$T_{PV} = { }T_{a} + { }\left( {{\mathrm{NOCT}} - { }25} \right) \times \frac{I}{800}$$

where, $I$ is the solar irradiance in W/m^2^, ${I}_{std}$ is the standard solar irradiance, ${R}_{c}$ is setting irradiance point of 120 W/m^2^, ${P}_{sm}$ is the maximum solar PV output power, ${P}_{r,STC}$ is the solar PV rated power at standard conditions STC, $\upgamma$ is power temperature coefficient in %/°C, ${T}_{PV}$ is the PV cell temperature, NOCT is nominal operating cell temperature which usually provided by each manufacturer but is approximately 45 ± 2 °C.

## Optimal power flow

This section presents the single objective OPF problem where, one objective function—such as total generation cost, carbon emissions, power losses, or voltage security index—is optimized under system constraints. In addition to the Multi-Objective OPF problem, more than one objective function is applied. In this paper, we focus on minimizing total cost (fuel plus renewable energy) and carbon emissions.

### Single objective OPF problem

In this problem, only one objective function is optimized at a time in single-objective optimization as explained in Eqs. (10), (11) and (12);

10 $$Min \;f(x)$$

Subject to:

11 $$s\left(x\right)=0$$

12 $$k\left(x\right)\le 0$$

where, *x* is the problem variables, $s\left(x\right)$ and $k\left(x\right)$ are the problem quality and inequality constraints respectively.

### Multi-objective OPF problem

Multi-objective optimization problem seeks to optimize multiple objectives at once, subject to a set of equality and inequality constraints that any feasible solution must satisfy. Formally, it can be stated in Eqs. (13–15)^40^.

13 $$Min \;{g}_{i}\left(x\right) i=\mathrm{1,2},\dots \dots ..,{N}_{obj}$$

Subject to:

14 $${s}_{i}\left(x\right)=o i=\mathrm{1,2},\dots \dots ..,{M}_{eq}$$

15 $${r}_{i}\left(x\right)\le o i=\mathrm{1,2},\dots \dots ..,{N}_{ineq}$$

where,${g}_{i}\left(x\right)$, is the mutli-objective function, *x* is is the number of objective functions, ${M}_{eq}$ and ${N}_{ineq}$ are the problem quality and inequality constraints respectively.

For a multi-objective optimization problem, a single global optimum is usually not sufficient to represent the solution, since improving one objective may lead to the degradation of another. Therefore, the concept of Pareto optimality is commonly used to identify a set of acceptable trade-off solutions. A solution is defined as Pareto optimal, non-dominated, or Pareto efficient if there is no other feasible solution that can improve at least one objective without worsening one or more of the remaining objectives^41^.

The Pareto front solutions are the dominant solutions over the whole search space. A fuzzy membership function ${\mu}_{i}$ is expressed in Eq. (16) to represent to represent the ${i}_{ih}$ objective function ${f}_{i}$. For each Pareto front $k$, the membership function ${\mu }^{k}$ is computed in Eq. (17). The maximum value of ${\mu }^{k}$ is the best compromise solution.

16 $$\mu _{i} = \left\{ {\begin{array}{*{20}l} 1 \hfill & {f_{i} \le f_{i} ^{{min}} } \hfill \\ {\frac{{f_{i} ^{{max}} - f_{i} }}{{f_{i} ^{{max}} - f_{i} ^{{min}} }}} \hfill & {f_{i} ^{{min}} < f_{i} < f_{i} ^{{max}} } \hfill \\ 0 \hfill & {f_{i} \ge f_{i} ^{{max}} } \hfill \\ \end{array} } \right.$$

17 $${\mu }^{k}=\frac{ {\sum}_{i=1}^{{N}_{obj}}{{\mu }^{k}}_{i}}{ \sum_{k=1}^{M}{\sum}_{i=1}^{{N}_{obj}}{{\mu }^{k}}_{i}}$$

### Objective functions

**(a) Fuel cost**

The goal of this objective function is to reduce the running costs of conventional thermal generators ${C}_{Th} (\mathrm{\$}/hr)$ using Eq. (18).

18 $$Min \;{C}_{Th}=\sum_{i=1}^{n}{a}_{i}+{b}_{i}{p}_{gi}+{c}_{i}{p}_{gi}^{2}$$

where, *n* is the thermal units number, (${a}_{i}$, ${b}_{i}$, ${c}_{i}$) are the coefficients of thermal units and ${p}_{gi}$ is the thermal unit generator’s active power.

**(b) Renewable energy generation cost**

The goal of this objective function is to reduce the running costs of both wind, ${C}_{w} (\mathrm{\$}/hr)$, and solar PV generation, ${C}_{s}(\mathrm{\$}/hr)$ using Eqs. (19) and (20).

19 $$Min \;{C}_{w}={C}_{R,w}+{C}_{P,w}$$

20 $$Min \;{C}_{s}={C}_{R,s}+{C}_{P,s}$$

where, ${C}_{R}$ and ${C}_{P}$ are the overestimation and underestimation costs, the initials w and s stand for wind and solar respectively.

When the available real renewable energy power (i.e. wind or solar) power is less than the planned power, electricity must be obtained from other sources, resulting in an increase in the operation cost known as overestimation cost ($${C}_{R})$$. If the actual available renewable energy power exceeds the planned amount, the operator will have to purchase additional electricity from the renewable energy farms that they did not expect and deal with it, a fee is known as underestimation cost ($${C}_{P})$$^50^. The costs of overestimating and underestimation are expressed in Eqs. (21) and (22).

21 $${C}_{R}={K}_{R}({{P}_{scheduled}-P}_{available})$$

22 $${C}_{P}={K}_{P}({P}_{available}-{P}_{scheduled})$$

where, ${K}_{R}$ and ${K}_{P}$ and are the overestimating and underestimation cost coefficients, respectively, ${P}_{scheduled}$ is the scheduled power, and ${P}_{available}$ is the available power by the renewable energy resource.

**(c) Carbon emissions**

The goal of this objective function is to reduce the carbon emissions from the thermal unit ${E}_{t}$ (ton/hr) as expressed in Eq. (23).

23 $$Min \;{E}_{t}=\sum_{i=1}^{n}{\alpha}_{i}+{\beta}_{i}{p}_{gi}+{\gamma}_{i}{p}_{gi}^{2}+{\xi}_{i}\mathrm{e}\mathrm{x}\mathrm{p}({\lambda}_{i}{p}_{gi})$$

where, ${\alpha}_{i}$, ${\beta}_{i}$, ${\gamma}_{i}$ and ${\xi}_{i}$ are the carbon emission coefficients of the thermal units.

**(d) Total cost including carbon tax**

Carbon tax is imposed to reduce energy production from thermal sources and increase reliance on renewable energy sources. Equation (24) shows the cost of carbon tax, ${C}_{Tax}$ due to carbon emissions from thermal units, ${E}_{t}$. Equation (25) illustrates the total cost ${C}_{T-1}(\mathrm{\$}/hr)$ including carbon tax^33^.

24 $${C}_{Tax}=CTR\times {E}_{t}$$

25 $$Min \;{C}_{T-1}={C}_{Th}+{C}_{w}+{C}_{s}+ {C}_{Tax}$$

where, $CTR$ is the carbon tax rate in $(\mathrm{\$}/ton)$.

**(e) Total cost including carbon tax and carbon credit**

To additionally encourage and increase the reliance on renewable energy, Carbon credit certificates are promoted for avoided emissions. These credits can then be sold or used to offset emissions elsewhere, making renewable energy both environmentally and financially beneficial. Hence, carbon credit will have a key contribution in reducing the total cost.

The offset carbon emissions are the emissions that are supposed to be generated by conventional thermal units. However, these emissions are avoided as the thermal units are replaced by renewable energy sources. Equation (26) is used to calculate the offset carbon emissions, ${E}_{offset} (ton/hr)$, Eqs. (27) and (28) illustrates the calculation of carbon credit revenue, ${CR}_{R}$, and the total cost ${C}_{T-2}(\mathrm{\$}/hr)$ including both carbon tax and carbon credit.

26 $${E}_{offset}=\sum_{i=1}^{k}{\alpha}_{i}+{\beta}_{i}{p}_{gi}+{\gamma}_{i}{p}_{gi}^{2}+{\xi}_{i}\mathrm{e}\mathrm{x}\mathrm{p}({\lambda}_{i}{p}_{gi})$$

27 $${CR}_{R}=CCR\times {E}_{offset}$$

28 $$Min \;{C}_{T-2}={C}_{Th}+{C}_{w}+{C}_{s}+ {C}_{Tax}-{CR}_{R}$$

where, *k* is the number conventional thermal that are replaced with renewable energy resources, $CCR$ is the carbon credit rate in $(\mathrm{\$}/ton)$

The dependent parameters, Active Power Losses and VSI can be calculated using Equations in^47^.

## Mayfly (MA) algorithm

The Mayfly Algorithm (MA) is a nature-inspired metaheuristic optimization technique based on the mating behavior and flight patterns of mayflies. It was proposed in 2020 by K. Zervoudakis and S. Tsafarakis, where, statistical and several benchmark tests and engineering problems were used showing the excellence of MA technique^50^. In the natural behavior of mayflies, a male mayfly (MM) performs a nuptial dance near a water surface to attract a female mayfly (FM), with mating typically occurring mid-air, followed by the deposition of eggs to continue the life cycle. Inspired by this process, the proposed Mayfly Algorithm (MA) models the optimization procedure through four main phases.

### Movment of MMs

MMs congregate in swarms around a body of water. This means that their position and movement speed are modified in response to the swarm’s other mayflies. The position of MMs can be formulated in Eq. (29). The velocity of mayfly can be calculated using Eq. (30).

29 $$x_{i}^{t + 1} = x_{i}^{t} + v_{i}^{t + 1}$$

30 $$v_{i}^{t + 1} = v_{i}^{t} + ae^{{ - \beta r_{p}^{2} }} \left( {pbest_{i} - x_{i}^{t} } \right) + be^{{ - \beta r_{g}^{2} }} \left( {gbest - x_{i}^{t} } \right)$$

where,${x}_{i}^{t}$ and ${x}_{i}^{t+1}$ are the current position and next position of MM number $i$, and ${v}_{i}^{t+1}$ is the MM velocity. *a* and *b* are postive constants, $pbest$ and $gbest$ are current best position and global best position, respectively, ${r}_{p}$ and ${r}_{g}$ are the distance between ${x}_{i}$ and current best position and global best position, respectively, $\beta$ is the fixed visibility coefficient.

### Movement of FMs

FMs, ${y}_{i}$, do not congregate in swarms; instead, they travel towards the male’s position in order to procreate. Equation (31) represents the change in FM postion.

31 $$y_{i}^{t + 1} = y_{i}^{t} + w_{i}^{t + 1}$$

where, ${w}_{i}^{t+1}$ represents the velocity of FMs that can be calculated using Eq. (32). The distance between FM and MM is represented by ${r}_{m}$.

32 $${w}_{i}^{t+1}={w}_{i}^{t}+b{e}^{-\beta {{r}_{m}}^{2}}\left({x}_{i}^{t}-{y}_{i}^{t}\right)$$

### Mating

The offspring are chosen in the same way that the FMs select their MMs for breeding. To develop and offspring, the best MM couples with the best FM. All MMs and FMs are ranked in the same way. Equations (33) and (34) explains the mayfly crossover.

33 $$offspring1 = L \times x_{i} + \left( {1 - L} \right) \times y_{i}$$

34 $$offspring2 = L \times y_{i} + \left( {1 - L} \right) \times x_{i}$$

where, *L* is a random number between 0 to 1.

### Mutation

The offspring are changed in order to keep the algorithm from becoming trapped on a local minimum which can be formulated using Eq. (35).

35 $${offspring}_{n}{\prime}={offspring}_{n}+{N}_{(\mathrm{0,1})}$$

where, ${N}_{(\mathrm{0,1})}$ is the normal distribution with mean = 0 and standard deviation = 1.

**Fig. 1:**
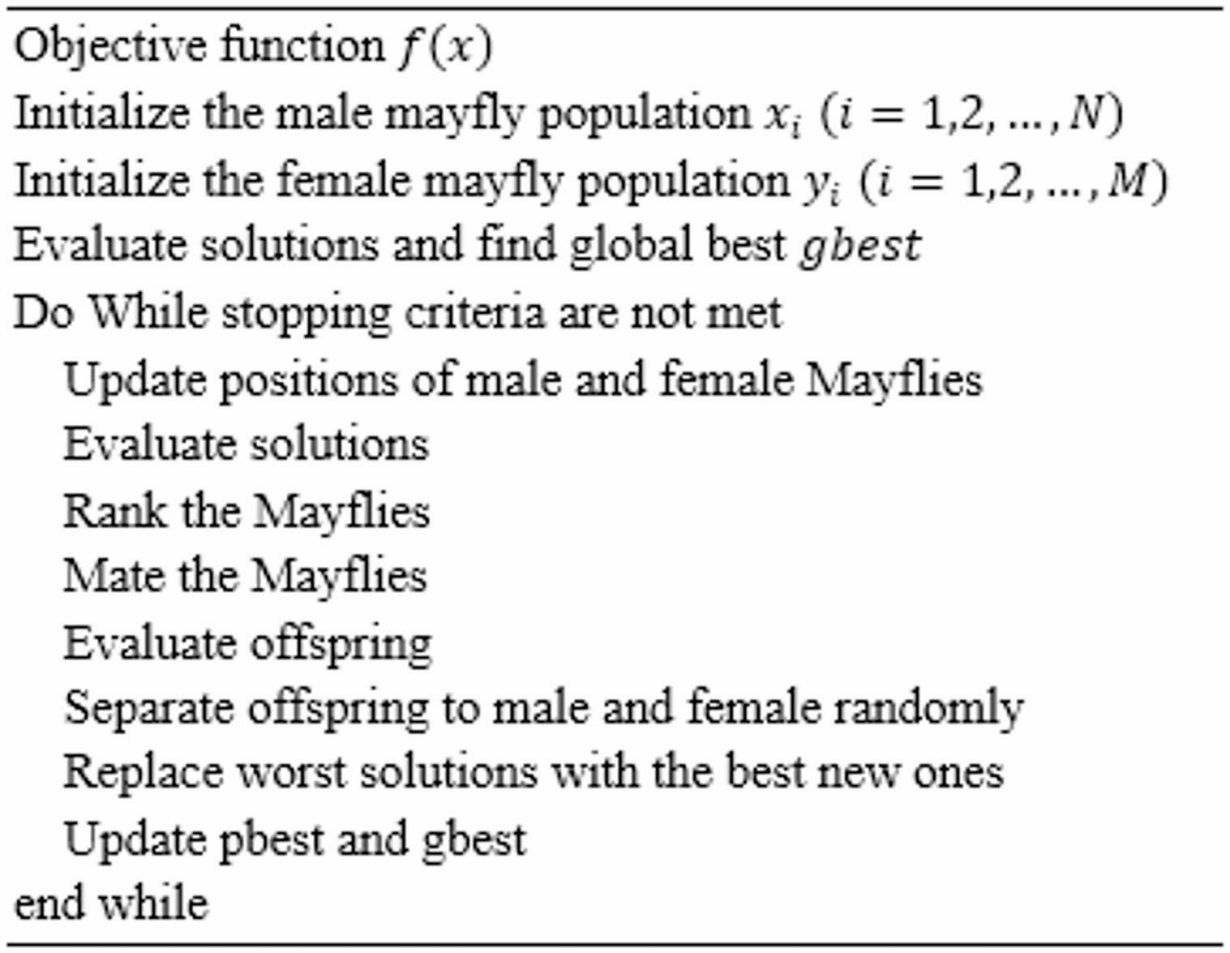
Pseudo code of the Mayfly algorithm.

The pseudo-code of the Mayfly algorithm is shown in Fig. 1. The maximum iteration number is 200 for solving OPF problem and population size of 20.

## Results and discussion

This work presents eleven case studies to demonstrate the stochastic modeling of wind and solar energy. First, MA algorithm is used to obtain the Weibull and Lognormal PDFs parameters for wind speed and solar irradiance modeling respectively. Second, MA is applied to study the classical OPF in the standard IEEE-30 bus system. Then SOPF problem under different scenarios is solved in a modified IEEE-30 bus system which contains 2 wind farms and 1 solar PV farm connected to buses 2, 5 and 13 respectively. The simulation case studies are summarized as follow:

- Case I: Wind Speed and Solar Irradiance Modeling for OPF Problem.
- Case II: Single Objective OPF.

Single objective OPF problem cases (with Ambient Temperature Effect):

- Case III: Single Objective SOPF: Total Cost Minimization including Carbon Credit and Carbon Tax.
- Case IV: Single Objective SOPF: Total Cost Minimization considering Ambient Temperature effect.
- Case V: Single Objective SOPF: Carbon Emissions Minimization considering Ambient Temperature effect.
- Case VI: Effect of Ambient Temperature When Minimizing Total Cost
- Case VII: Effect of Ambient Temperature When Minimizing Carbon Emissions.

Multi-period single objective OPF problem cases (with Ambient Temperature Effect and Renewable Energy Degradation):

- Case VIII: Multi-Period Single Objective SOPF: Total Cost Minimization considering Ambient Temperature and Degradation effects.
- Case IX: Effect of Renewable Energy Degradation and Ambient Temperature on Total Cost and Carbon Emissions.

Multi-objectiveSOPF problem cases (with Ambient Temperature Effect and Renewable Energy Degradation):

- Case X: Multi-Objective SOPF considering Ambient Temperature (No Degradation)..
- Case XI: Multi-Period Multi-Objective SOPF considering Ambient Temperature and Degradation effects.

The Newton–Raphson technique was used to calculate power flow using the MATPOWER program.

### Case I: wind speed and solar irradiance modeling for OPF problem

**Fig. 2:**
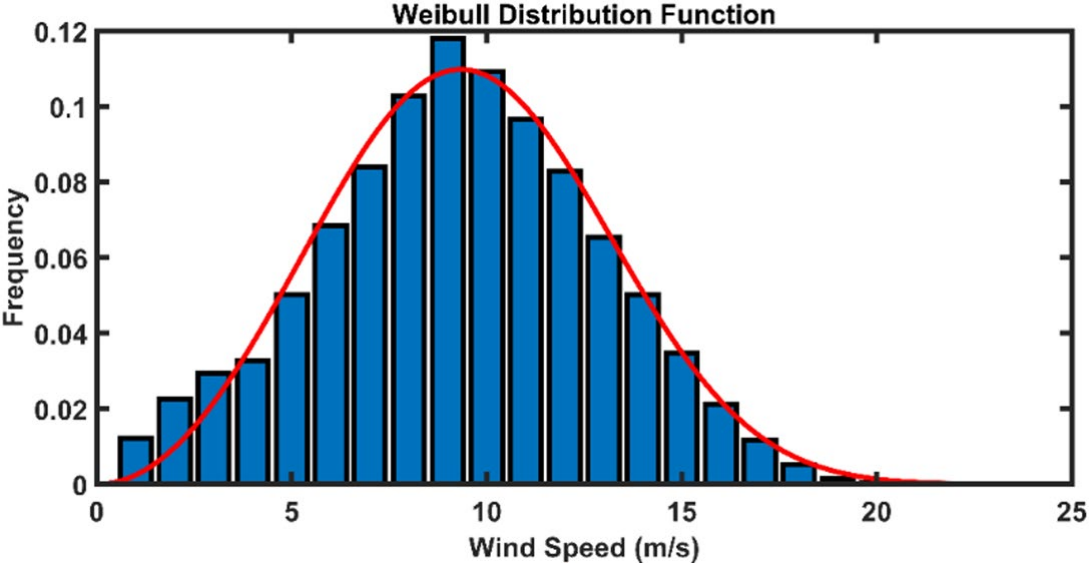
Wind speed modeling.

**Fig. 3:**
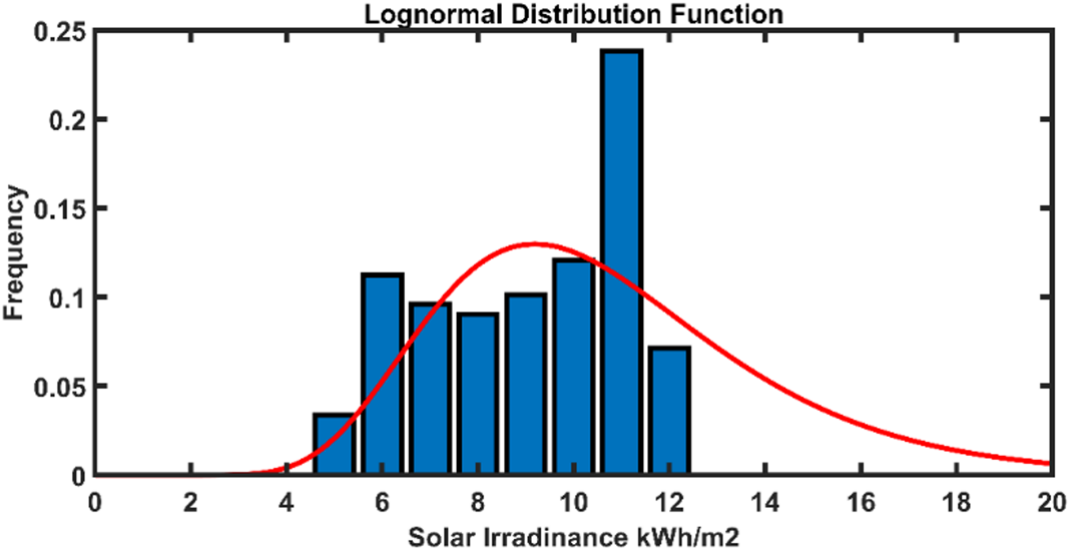
Solar irradiance modeling.

Based on Literature Survey, Weibull PDF is selected to model wind speed data while, Lognormal PDF is selected to model solar iradiance date. In this case study, MA is used to estimate the Weibull distribution parameters to model 3-year wind speed data. In addition to estimating the Lognormal distribution parameters for 5-year solar irradiance data. For accurate modeling, the objective of minimalizing root mean square error (RMSE) and Coefficient of Correlation (R^2^) are used as judging criteria as explained in Eqs. (36) and (37). Figures 2 and 3 show the fitting of both Weibull and Lognormal PDFs respectively. Table 1 shows the PDFs parameter and the judjing criteria.

36 $$RMSE=\sqrt{\frac{1}{2} \sum_{i=1}^{k}{({y}_{i}-{x}_{i})}^{2}}$$

37 $${R}^{2}= \frac{\sum_{i=1}^{k}{({y}_{i}-m)}^{2}-\sum_{i=1}^{k}{({x}_{i}-m)}^{2}}{\sum_{i=1}^{k}{({y}_{i}-m)}^{2}}$$

where, n represents the number of data classes, ${y}_{i}$ represents the i-th data from real data, ${x}_{i}$ represents the predicted i-th data and *m* represents the average data. In this case the data is wind speed and solar irradiance.

**Table 1:** Weibull parameters and judging criteria.

	Wind speed weibull PDF	Solar irradiance lognormal PDF
PDF parameters	k = 2.995 c = 10.69	$\upmu$= 2.21 σ = 0.27
RMSE	0.00506	0.042
R^2^	0.9836	0.958

### Case II: single objective OPF

**Fig. 4:**
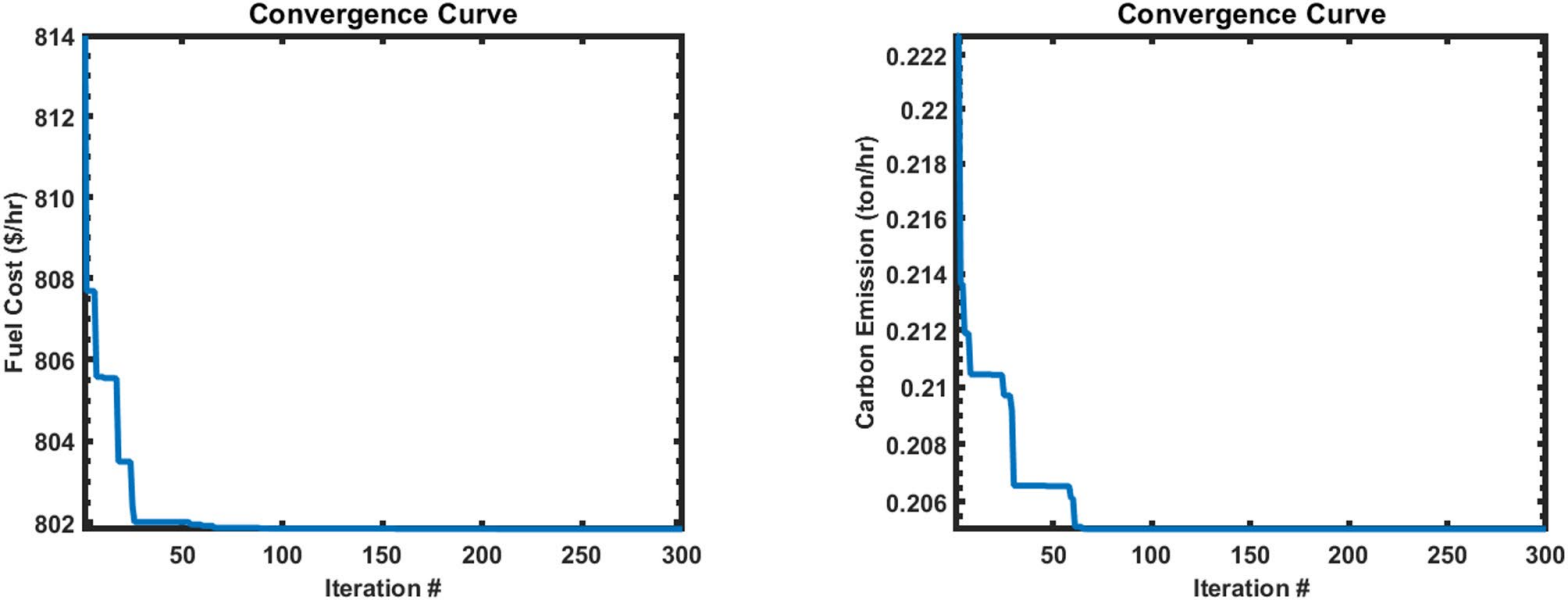
Convergence curves for fuel cost and carbon emission minimization.

In this case study, two single objective OPF are deployed to minimize fuel cost and Carbon Emission using the EDO technique in standard IEEE-30 bus system. The convergence curves for fuel cost and carbon emission are shown in Fig. 4. The optimal control variable and optimal results are shown in Table 2 and compared to multiple algorithms showing the excellence of MA algorithm.

**Table 2:** Comparison of fuel cost and carbon emissions minimization.

Method	Fuel cost minimization ($/hr)	Method		Carbon emissions minimization(Ton/hr)
MA	801.84	MA		0.205
GA^40^	801.96	GA^40^		0.20723
GWO^40^	801.86	PSO^52^		0.2063
ALO^23^	801.85	IPSO^52^		0.2058
SSA^23^	801.86	MPSO-SFLA^53^		0.2052
PSO^54^	802	GSO^54^		0.206
DA-PSO^54^	802.12	AGSO^54^		0.2059

### Case III: single objective SOPF: total cost minimization including carbon credit and carbon tax

**Fig. 5:**
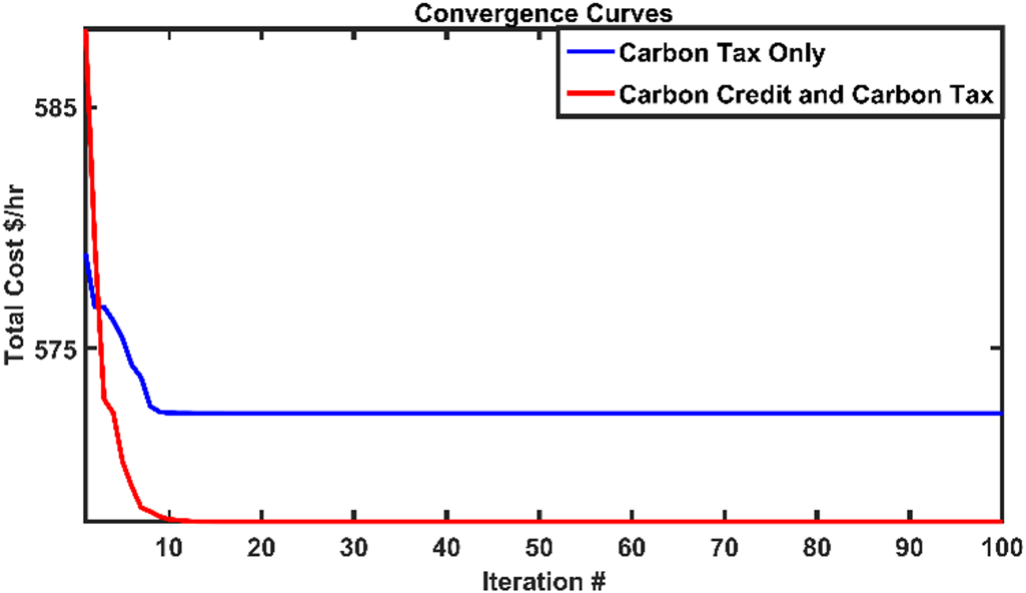
Convergence curves for carbon tax and carbon credit.

MA is applied to the modified IEEE‑30 bus system to determine the optimal scheduled power, where buses 2 and 5 are converted into wind farms rated at 80 MW and 50 MW, respectively while bus 13 is converted to solar PV farm with 40MW rated capacity. This case is to validate the effect of add carbon credit on the objective of total cost minimization compared to the case when only carbon tax is considered in the same objective function. The Weibull and Lognormal PDF parameters for wind speed and solar irradiance are obtained in Table 2. The reserve cost coefficient is ${K}_{R}=4$ and Penalty cost coefficient ${K}_{P}=1$ for wind while for solar ${K}_{R}=20$ and Penalty cost coefficient ${K}_{P}=1.5$. the carbon tax rate,$CTR$, is 20 $(\mathrm{\$}/ton)$^42^ while the carbon credit rate, $CCR$, is 30 $(\mathrm{\$}/ton)$^55^. Figure 5 shows the convergence curves when carbon tax is only considered as well as when both carbon tax and carbon credit are both considered in total operation cost minimization respectively. Table 3 shows the optimal control variable and optimal results.

**Table 3:** The optimal control variables considering carbon tax and credit.

Parameter	With carbon tax only	With carbon tax and credit
${P}_{G1}$	127.0165	126.893
${P}_{2-wind}$	69.115	69.327
${P}_{5-wind}$	44.635	44.578
${P}_{G8}$	10	10
${P}_{G11}$	10	10
${P}_{13-PV}$	29.267	29.236
Total cost	**572.474**	**568.84**
Fuel cost	380.362	379.98
Wind power cost	156.55	156.942
Solar power cost	32.096	30.6
Power loss	6.634	6.633
VSI	7.432	7.433
Carbon emission	**0.1732**	**0.1729**

Bold for the objective function in the optimization.

The results demonstrate that when both carbon credit and carbon tax are included in the total operation cost objective function, the total cost is reduced by 0.8% compared to the case when carbon tax is only included. Accordingly, carbon credit increases the reliance on renewable energy.

**Fig. 6:**
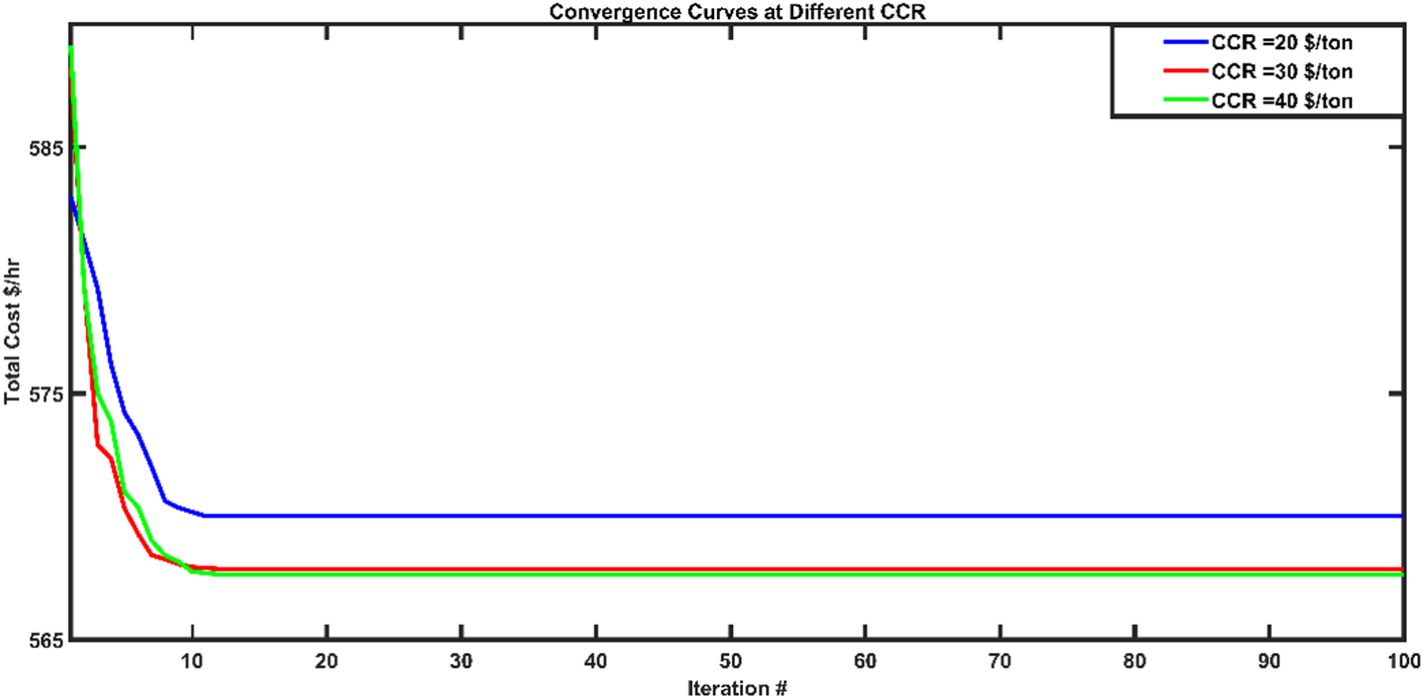
Convergence curves for carbon tax and carbon credit.

Table 4 evaluates the impact of different carbon charge rates on the performance of the proposed SOPF problem. By considering different carbon-pricing levels, the analysis demonstrates how the carbon-credit mechanism influences the total operating cost, emission-related cost, and optimal dispatch decisions. The obtained results confirm that incorporating carbon credits remains effective under different charge-rate assumptions, as it consistently encourages lower-emission operation. Figure 6 shows the convergence curves at different carbon credit rates.

**Table 4:** The optimal control variables at different carbon credit rates.

Parameter	CCR (20 $/ton)	CCR (30 $/ton)	CCR (40 $/ton)
$${P}_{G1}$$	127.142	126.893	126.684
$${P}_{2-wind}$$	69.154	69.327	69.4
$${P}_{5-wind}$$	44.573	44.578	44.68
$${P}_{G8}$$	10	10	10
$${P}_{G11}$$	10	10	10
$${P}_{13-PV}$$	29.174	29.236	29.26
Total cost	**570.02**	**568.83**	**567.64**
Fuel cost	380.732	379.98	379.38
Wind power cost	157.7524	156.942	158.27
Solar power cost	29.953	30.6	30.304
Power loss	6.6416	6.633	6.623
VSI	7.433	7.433	7.4328
Carbon emission	**0.1731**	**0.1729**	**0.1727**

Bold for the objective function in the optimization.

The results indicate that the total operating cost decreases progressively as the carbon credit rate (CCR) increases. At a CCR of 20 $/ton, the total cost is 570.02 $/h, while increasing the CCR to 30 $/ton reduces the cost to 568.83 $/h. This corresponds to a cost saving of 1.19 $/h, equivalent to approximately **0.21%**. When the CCR is further increased to 40 $/ton, the total cost decreases to 567.64 $/h, resulting in a total saving of 2.38 $/h, or about **0.42%**, relative to the 20 $/ton case. These results confirm that higher CCR values enhance the economic effectiveness of the proposed carbon-credit mechanism.

### Case IV: single objective SOPF: total cost minimization considering ambient temperature effect

**Fig. 7:**
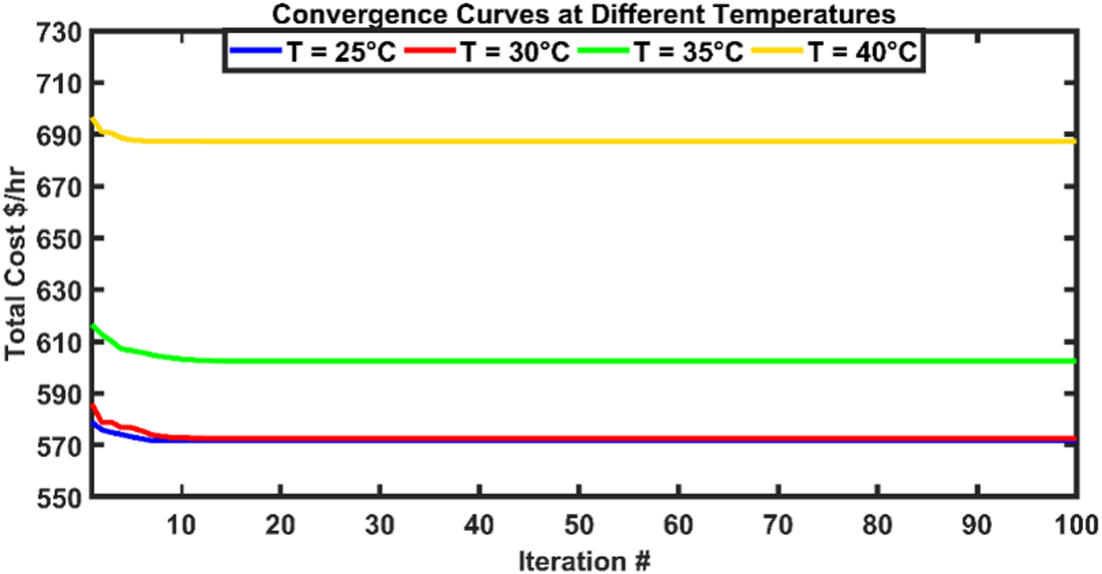
Convergence curves at different temperatures.

The MA is used to solve SCOPF in a modified IEEE-30 bus system when the ambient temperature is taken into account in the range of 25–40 °C with 5 °C. step. The objective function is total cost minimization including both carbon tax and carbon credit. To calculate the wind maximum output power, Gamesa G80 wind turbine is used with ${v}_{cut-in}$ = 3.5 m/s, ${v}_{r}$ = 12 m/s and ${v}_{cut-out}$ = 25 m/s and de-rating curve is described in^46^ where, the wind turbine de-rating starts when the ambient temperature exceeds 30 °C. While to obtain solar PV output power, Eqs. (7–9) are used with $\upgamma$ = −0.4%/°C and NOCT = 45 °C^49^. Figure 7 shows the convergence curves for the objective function. Table 5 shows the optimal control variable and optimal results at different ambient temperatures

**Table 5:** The optimal control variables at different temperatures.

Parameter	$25^\circ C$	$30^\circ C$	$35^\circ C$	$40^\circ C$
${P}_{G1}$	129.223	129.793	144.767	171.33
${P}_{2-wind}$	69.622	69.584	61.128	37.545
${P}_{5-wind}$	44.793	44.71	39.3658	24.454
${P}_{G8}$	10	10	10	21.33
${P}_{G11}$	10	10	10	12.181
${P}_{13-PV}$	26.593	26.004	25.617	25.23
Total cost	**571.72**	**572.691**	**602.516**	**687.182**
Fuel cost	386.9	388.59	433.954	566.083
Wind power cost	156.983	158.021	143.365	95
Solar power cost	27.166	24.4	24.015	23.97
Power loss	6.749	6.774	7.477	8.67
VSI	7.459	7.465	7.464	7.456
Carbon emission	0.1759	0.1768	0.2	0.2504

Bold for the objective function in the optimization.

### Case V: single objective SOPF: carbon emissions minimization considering ambient temperature effect

**Fig. 8:**
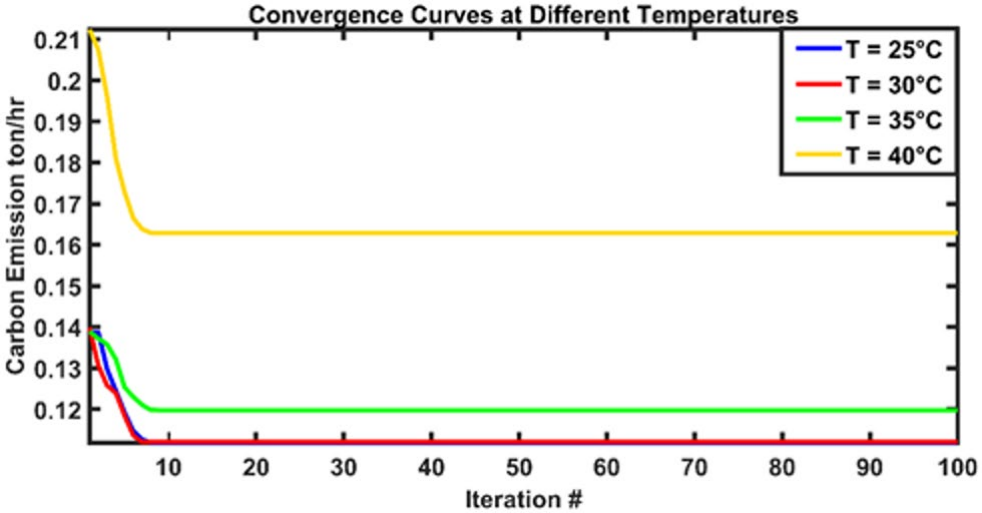
Convergence curves for carbon emission minimization.

In this case, the objective function for SOPF is to minimize carbon emissions. Figure 8 shows the convergence curves for the objective function. Table 6 shows the optimal control variable and optimal results at different ambient temperatures.

**Table 6:** Control variables and optimal results carbon emission minimization at different ambient temperatures.

Parameter	$25^\circ C$	$30^\circ C$	$35^\circ C$	$40^\circ C$
${P}_{G1}$	56.986	57.802	78.655	126.86
${P}_{2-wind}$	80	80	68	40
${P}_{5-wind}$	50	50	42.5	25
${P}_{G8}$	35	35	35	35
${P}_{G11}$	30	30	30	30
${P}_{13-PV}$	35	34.2	33.4	32.6
Total cost	664.685	665.615	676.75	728.974
Fuel cost	362.567	364.55	416.93	550.483
Wind power cost	212.074	213.37	173.87	95.62
Solar power cost	90.045	87.7	85.952	82.87
Power loss	3.5857	3.6017	4.1552	6.0583
VSI	7.26	7.267	7.267	7.259
Carbon emission	**0.11183**	**0.112**	**0.1198**	**0.1629**

Bold for the objective function in the optimization.

### Case VI: effect of ambient temperature when minimizing total cost

In this part, the impact of the ambient temperature is discussed in detail. The percentage of change of any optimal results can be calculated using Eq. (38).

38 $$\Delta X= \frac{{X}_{Tamb}-{X}_{base}}{{X}_{ base}} \%$$

where, *X* is any optimal results (ex: fuel cost, carbon emission,..etc.), ${X}_{Tamb}$ is the result at Temperature in °C and ${X}_{base}$ is the result when the temperature effect is ignored in case III.

**(a) Effect on total operation cost**

**Fig. 9:**
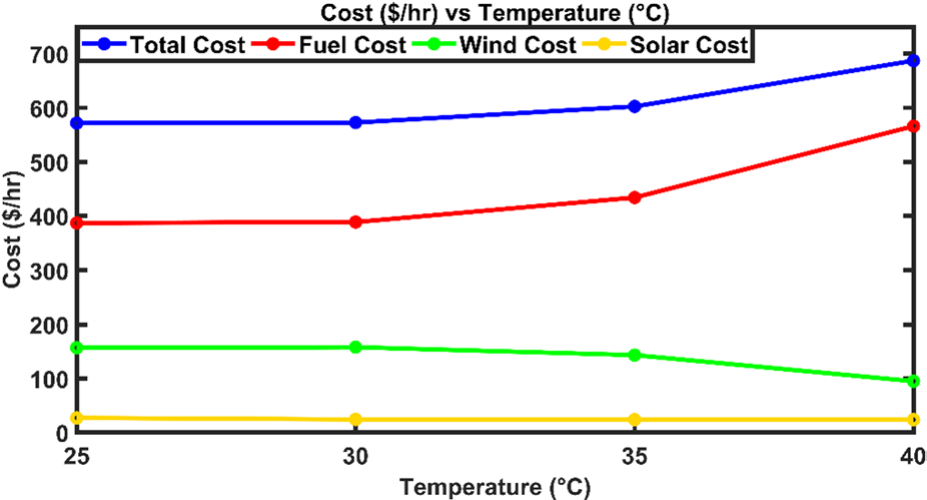
Cost variation with ambient temperature.

Figure 9 shows how the total cost, fuel cost and wind cost varies with the change of the ambient temperature. Table 7 shows the percentage of change of the total cost and fuel cost.

**Table 7:** Percentage of change of the total cost and fuel cost.

Parameter	$25^\circ C$	$30^\circ C$	$35^\circ C$	$40^\circ C$
Δ Total cost %	0.68%	0.85%	6.1%	21.01%
Δ Fuel cost %	2.44%	2.89%	14.9%	49.9%

It can be concluded from the table, At higher ambient temperatures, the cost impact becomes more significant and has important planning implications. At 35 °C, the total cost increases by 6.1% and the fuel cost by 14.9%, while at 40 °C the increase reaches 21.01% and 49.9%, respectively. These results indicate that high-temperature conditions can reduce renewable contribution and increase dependence on thermal generation, which should be considered in long-term generation and operation planning. To mitigate these impacts, planners may consider energy storage integration, demand response, reserve capacity to enhance system flexibility under stressed climate conditions.

**(b) Effect on carbon emissions**

**Fig. 10:**
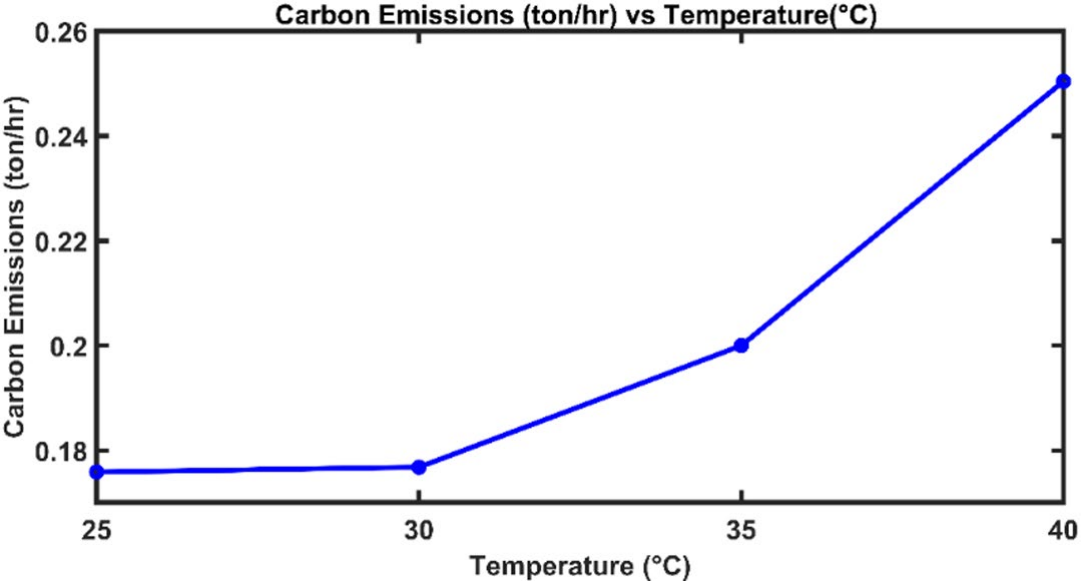
Cost Variation with Ambient Temperature.

Figure 10 shows how the carbon emission vary with the change of the ambient temperature. Table 8 shows the percentage of change of the carbon emissions.

**Table 8:** Percentage of change of carbon emissions.

Parameter	$25^\circ C$	$30^\circ C$	$35^\circ C$	$40^\circ C$
Δ emissions %	2.33%	2.9%	16.35%	45.67%

It can be deduced from the table, At elevated ambient temperatures, the emission impact becomes highly significant. At 35 °C, emissions increase by 16.35%, while at 40 °C the increase reaches 45.67%. This indicates that high-temperature conditions reduce renewable generation effectiveness and require greater thermal generation dispatch, leading to higher carbon emissions. From a planning perspective, these results highlight the need to incorporate climate-related derating in long-term emission assessments and to consider mitigation measures such as energy storage, higher renewable reserve margins, low-carbon backup generation to limit emission increases under stressed temperature conditions.

### Case VII: effect of ambient temperature when minimizing carbon emissions

**(a) Effect on total operation cost**

**Fig. 11:**
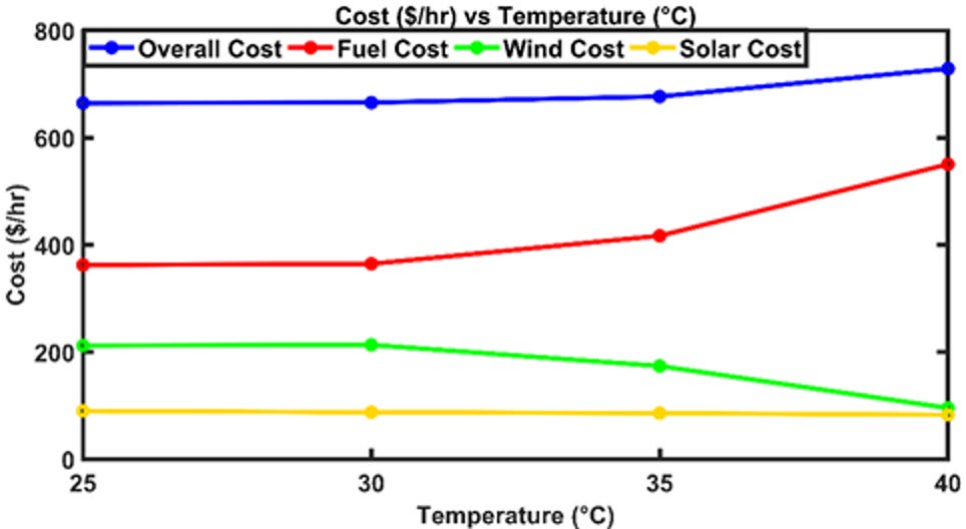
Cost variation with ambient temperature.

Figure 11 shows how the total cost, fuel cost and wind cost varies with the change of the ambient temperature. Table 9 shows the percentage of change of the total cost and fuel cost.

**Table 9:** Percentage of change of the total operation cost, fuel cost and wind cost.

Parameter	$30^\circ C$	$35^\circ C$	$40^\circ C$
Δ Total Cost %	0.14%	1.82%	9.67%
Δ Fuel Cost %	0.547%	15%	51.83%

It can be concluded from the table, At higher ambient temperatures, the cost impact becomes more significant and has important planning implications. At 35 °C, the total cost increases by 1.82% and the fuel cost by 15%, while at 40 °C the increase reaches 9.67% and 51.83%, respectively. These results indicate that high-temperature conditions can reduce renewable contribution and increase dependence on thermal generation, which should be considered in long-term generation and operation planning. To mitigate these impacts, planners may consider energy storage integration, demand response, reserve capacity to enhance system flexibility under stressed climate conditions.

**(b) Effect on carbon emissions**

**Fig. 12:**
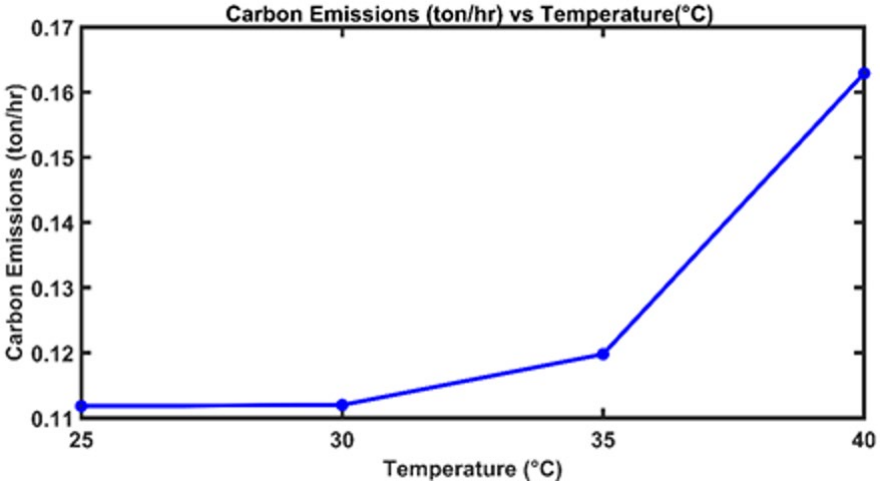
Carbon emission variation with ambient temperature.

Figure 12 shows how the carbon emission vary with the change of the ambient temperature. Table 10 shows the percentage of change of the carbon emissions.

**Table 10:** Percentage of change of carbon emissions.

Parameter	$30^\circ C$	$35^\circ C$	$40^\circ C$
Δ emissions %	0.15%	7.13%	45.7%

It can be deduced from the table, At elevated ambient temperatures, the emission impact becomes highly significant. At 35 °C, emissions increase by 7.13%, while at 40 °C the increase reaches 45.7%. This indicates that high-temperature conditions reduce renewable generation effectiveness and require greater thermal generation dispatch, leading to higher carbon emissions. From a planning perspective, these results highlight the need to incorporate climate-related derating in long-term emission assessments and to consider mitigation measures such as energy storage, higher renewable reserve margins, low-carbon backup generation to limit emission increases under stressed temperature conditions.

### Case VIII: multi-period single objective SOPF: total cost minimization considering ambient temperature and degradation effects

Renewable-generation degradation directly affects the long-term performance of PV modules and wind turbines by gradually reducing their effective power output over the project lifetime. As the equipment ages, annual energy production decreases, which may reduce renewable penetration and require higher dispatch from conventional generators. This reduction can also increase operating cost and carbon emissions. In this case, Multi-period Single Objective SOPF is solved to minimize the total cost considering the degradation factor of renewable energy resources during the lifetime as well as the ambient temperature effect to impact of both degradation and ambient temperature on the total cost and carbon emissions during the lifetime of renewable energy projects.

Equation (39) shows how the output from renewable energy either wind or solar is reduced over time due to degradation.

39 $${P(o)}_{w/s}= {Pm}_{w/s} x {(1-DF)}^{n}$$

where, ${P(t)}_{w/s}$ is the degraded power of either wind or solar after n-years, ${Pm}_{w/s}$ is the maximum output power either from wind or solar at the beginning of operation (i.e. n = 0), $DF$ is the degradation factor in %/year.

The same conditions in this case is maintained as case IV, the lifetime of wind and solar PV farms is considered as 25 years, the degradation factor, $DF$, is assumed 0.6%^56,57^. Cases II–VII considers only the solution of SOPF problem in the beginning of operation. Hence, the multi-period SOPF problem is solved from the beginning of operation till the end of lifetime with 5-year step considering both degradation and ambient temperature effects.

**Fig. 13:**
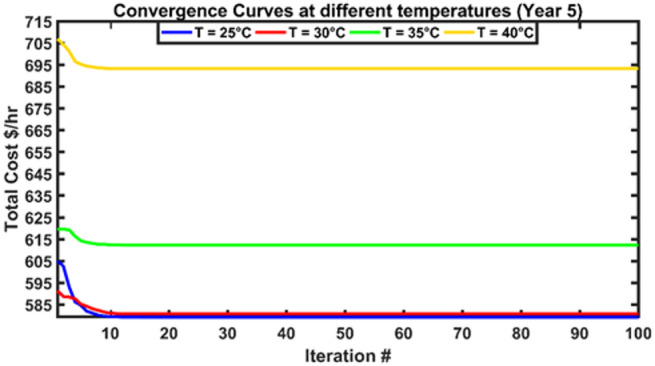
Convergence curves at different temperatures after 5 years.

**Fig. 14:**
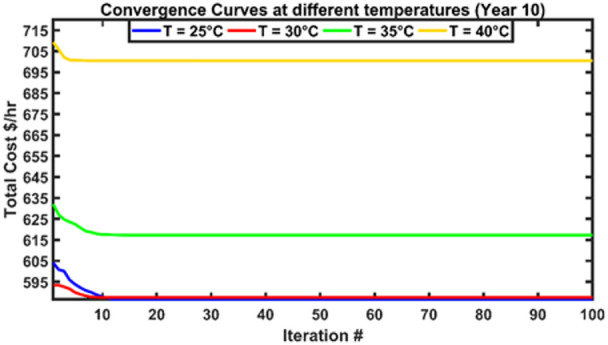
Convergence curves at different temperatures after 10 years.

**Fig. 15:**
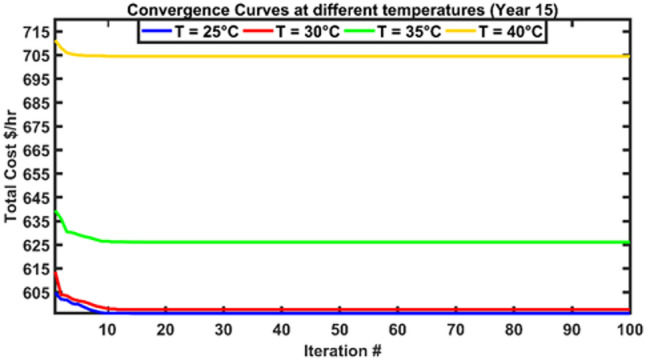
Convergence curves at different temperatures after 15 years.

**Fig. 16:**
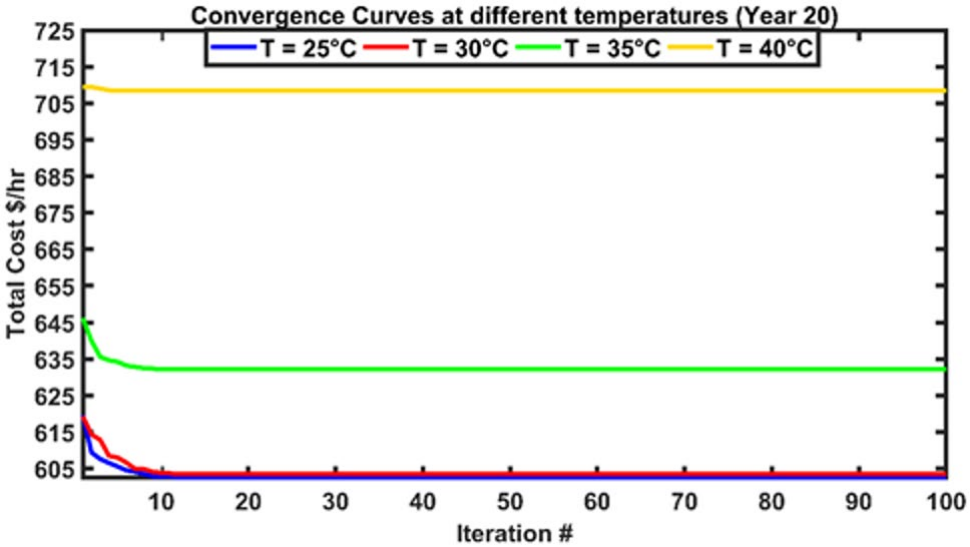
Convergence curves at different temperatures after 20 years.

**Fig. 17:**
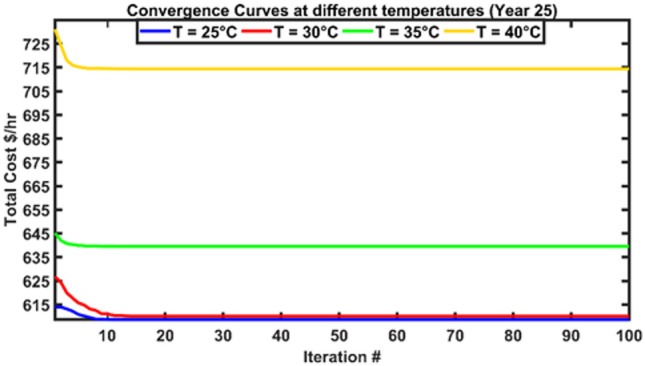
Convergence curves at different temperatures after 25 years.

Figures 13, 14, 15, 16 and 17 show the convergence curves for the objective function. Tables 11 and 12 show the optimal control variable and optimal results at different years and different ambient temperatures.

**Table 11:** Optimal Results at different loading conditions.

Case	Overall cost ($/hr)	Fuel cost ($/hr)	Wind power cost ($/hr)	Solar cost ($/hr)	Carbon emissions (Ton/hr)
Year 5					
At ${T}_{amb}=25^\circ C$	579.2651	398.4892	156.0094	23.9806	0.18143
At ${T}_{amb}=30^\circ C$	580.8583	399.8533	156.5572	23.6544	0.18208
At ${T}_{amb}=35^\circ C$	612.2912	445.2777	142.7195	22.9867	0.20583
At ${T}_{amb}=40^\circ C$	693.3613	574.571	93.2858	23.3258	0.25371
Year 10					
At ${T}_{amb}=25^\circ C$	586.5805	408.2518	154.3111	23.133	0.1861
At ${T}_{amb}=30^\circ C$	587.5284	409.3105	154.2019	23.1255	0.1867
At ${T}_{amb}=35^\circ C$	617.3146	452.6764	140.297	22.9736	0.21
At ${T}_{amb}=40^\circ C$	700.5074	582.9629	92.6471	22.6681	0.257
Year 15					
At ${T}_{amb}=25^\circ C$	596.0639	420.7201	152.0163	22.3115	0.1925
At ${T}_{amb}=30^\circ C$	597.639	421.214	152.9598	22.4507	0.19275
At ${T}_{amb}=35^\circ C$	626.1256	465.4567	136.9626	22.2546	0.2135
At ${T}_{amb}=40^\circ C$	704.5662	590.1078	89.629	22.5573	0.26
Year 20					
At ${T}_{amb}=25^\circ C$	602.6104	430.1931	148.9089	22.3923	0.1975
At ${T}_{amb}=30^\circ C$	603.5319	431	149.7269	21.6894	0.198
At ${T}_{amb}=35^\circ C$	632.1516	477.2942	131.8856	21.4391	0.2178
At ${T}_{amb}=40^\circ C$	708.3945	599.04	85.772	21.2546	0.264
Year 25					
At ${T}_{amb}=25^\circ C$	608.9237	438.6985	147.6068	21.4095	0.2021
At ${T}_{amb}=30^\circ C$	610.0805	440.3671	147.4655	21.0256	0.2031
At ${T}_{amb}=35^\circ C$	639.5533	486.1238	130.5892	21.2492	0.221
At ${T}_{amb}=40^\circ C$	714.4659	606.4036	84.9081	20.781	0.267

**Table 12:** Control variables at different loading conditions.

Case	${P}_{G1}$(MW)	${P}_{2-wind}$(MW)	${P}_{5-wind}$(MW)	${P}_{G8}$(MW)	${P}_{G11}$(MW)	${P}_{13-PV}$(MW)
Year 5						
At ${T}_{amb}=25^\circ C$	133.1086	67.70499	43.70534	10	10	25.8
At ${T}_{amb}=30^\circ C$	133.5633	67.80545	43.71127	10	10	25.26471
At ${T}_{amb}=35^\circ C$	148.4203	59.40394	38.42151	10	10	24.81289
At ${T}_{amb}=40^\circ C$	172.7655	36.5906	23.76023	22.10592	12.44582	24.47344
Year 10						
At ${T}_{amb}=25^\circ C$	136.3514	66.26561	42.77348	10	10	25.08158
At ${T}_{amb}=30^\circ C$	136.7015	66.37732	42.73282	10	10	24.68222
At ${T}_{amb}=35^\circ C$	150.2814	58.47231	37.8	10.465	10	24.125
At ${T}_{amb}=40^\circ C$	174.1811	35.52027	23.31763	22.8716	12.7061	23.605
Year 15						
At ${T}_{amb}=25^\circ C$	140.4555	64.35368	41.52	10	10	24.34
At ${T}_{amb}=30^\circ C$	140.6173	64.60212	41.69448	10	10	23.76655
At ${T}_{amb}=35^\circ C$	152.8584	56.66378	36.57143	11.83273	10	23.32441
At ${T}_{amb}=40^\circ C$	175.3801	34.75121	22.7	23.52255	12.928	22.985
Year 20						
At ${T}_{amb}=25^\circ C$	143.5462	63.01935	40.6498	10	10	23.608
At ${T}_{amb}=30^\circ C$	143.807	63.09463	40.85	10	10	23.085
At ${T}_{amb}=35^\circ C$	155.2342	54.83584	35.43153	13.08858	10	22.7574
At ${T}_{amb}=40^\circ C$	176.88	33.565	21.9	24.3277	13.205	22.465
Year 25						
At ${T}_{amb}=25^\circ C$	146.3015	61.76225	39.9	10	10	23
At ${T}_{amb}=30^\circ C$	146.84	61.8	39.86	10	10	22.5
At ${T}_{amb}=35^\circ C$	157	53.6317	34.683	14.027	10	22.09
At ${T}_{amb}=40^\circ C$	178.1042	32.791	21.4	25	13.433	21.682

### Case IX: effect of renewable energy degradation and ambient temperature on total cost and carbon emissions

**(a) Effect on total operation cost**

**Fig. 18:**
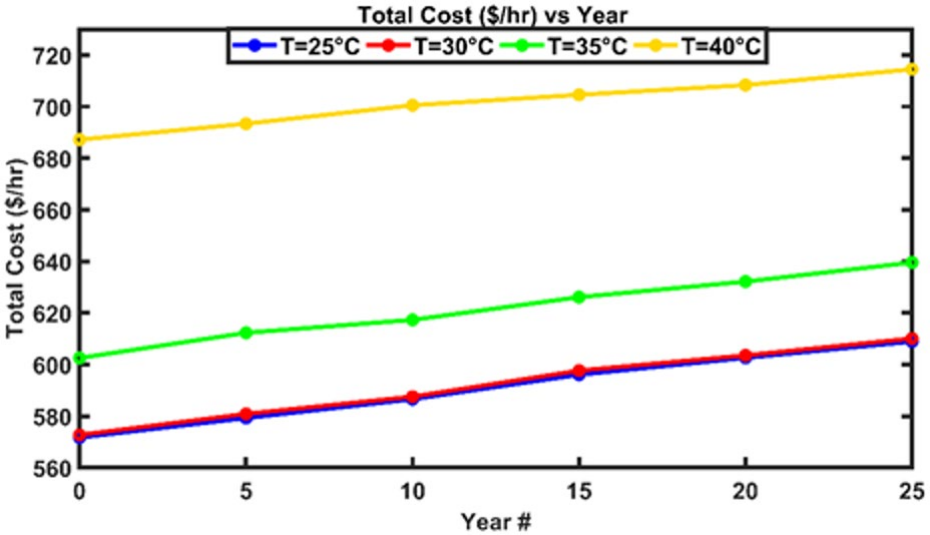
Total cost variation with ambient temperature after 25 years.

**Fig. 19:**
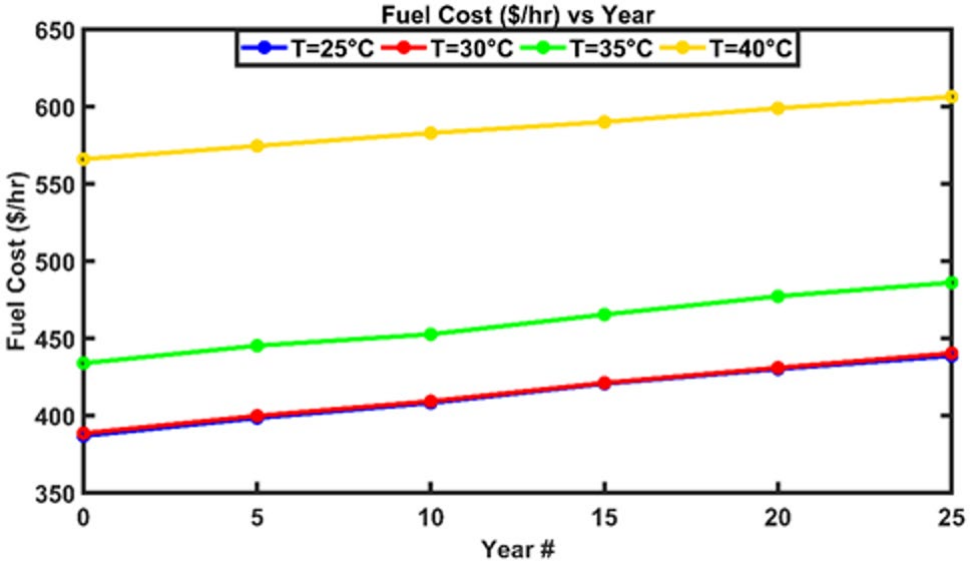
Total cost variation with ambient temperature after 25 years.

Figures 18 and 19 show how the total cost varies with the number of years during the operation of the wind and solar PV plants at different ambient temperatures. Table 13 shows the percentage of change of the total cost and fuel cost at the end of the project life time (25 years) compared to the beginning of when the ambient temperature increases from 25 to 40 °C (Case IV).

**Table 13:** Percentage of change of the Total operation cost, Fuel cost and Wind cost.

Parameter	$30^\circ C$	$35^\circ C$	$40^\circ C$
Δ Total cost %	6.71%	11.86%	24.96%
Δ Fuel cost %	13.82%	25.64%	56.73%

It can be concluded from the table, the total cost increased by 24.96% when the ambient temperature changes from 25 to 40 °C after 25 years, while the fuel cost increased by 56.73% as a result of reduction in scheduled power due to both degradation and temperature effect.

**(b) Effect on carbon emissions**

**Fig. 20:**
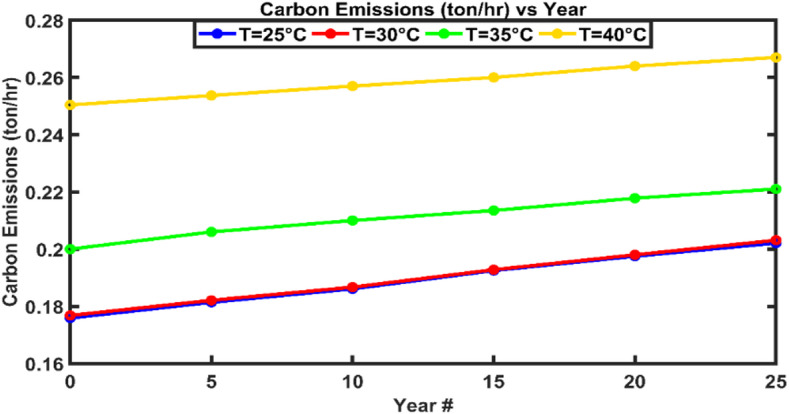
Variation of carbon emissions with years of operation at different ambient temperatures.

Figure 20 shows how the carbon emissions vary with the number of years during the operation of the wind and solar PV plants at different ambient temperatures. Table 14 shows the percentage of change of the carbon emissions at the end of the project life time (25 years) compared to the beginning at different ambient temperatures.

**Table 14:** Percentage of change of carbon emissions.

Parameter	$30^\circ C$	$35^\circ C$	$40^\circ C$
Δ Emissions %	15.46%	25.6%	51.8%

It can be concluded from the carbon emissions increased by 51.8% when the ambient temperature changes from 25 to 40 °C after 25 years, as a result of reduction in scheduled power and increasing the reliance on conventional thermal plants considering both degradation and temperature effect.

### Case X: multi-objective SOPF considering ambient temperature (no degradation)

**Fig. 21:**
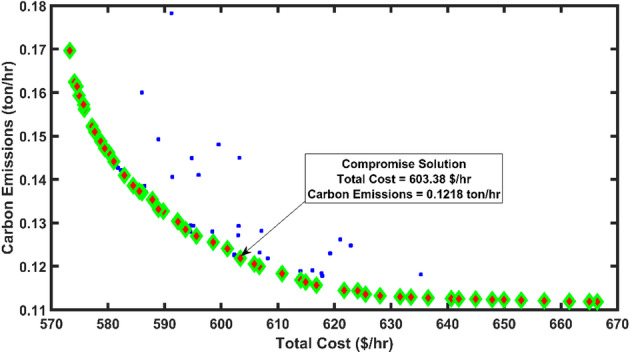
Pareto optimal solutions and the best obtained compromise solution at 25 °C.

**Fig. 22:**
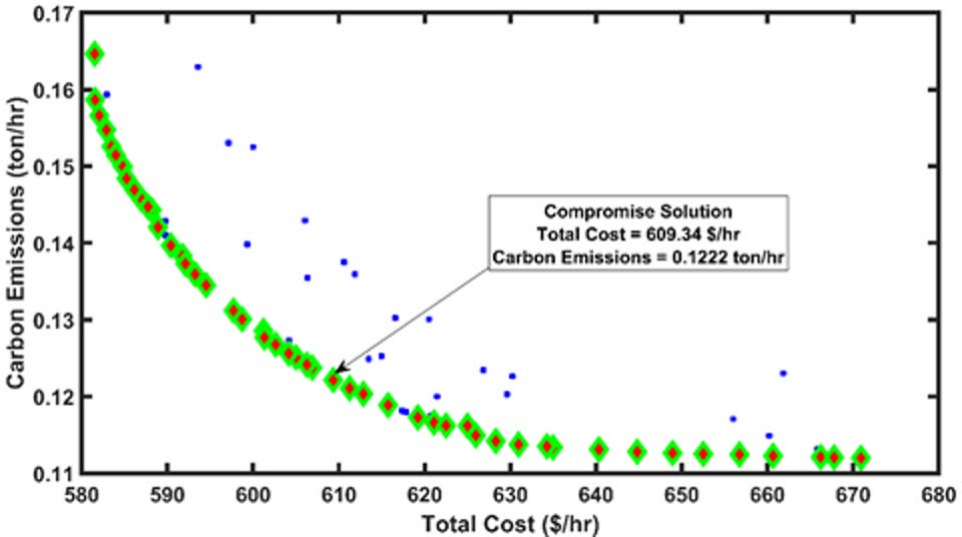
Pareto optimal solutions and the best obtained compromise solution at 30 °C.

**Fig. 23:**
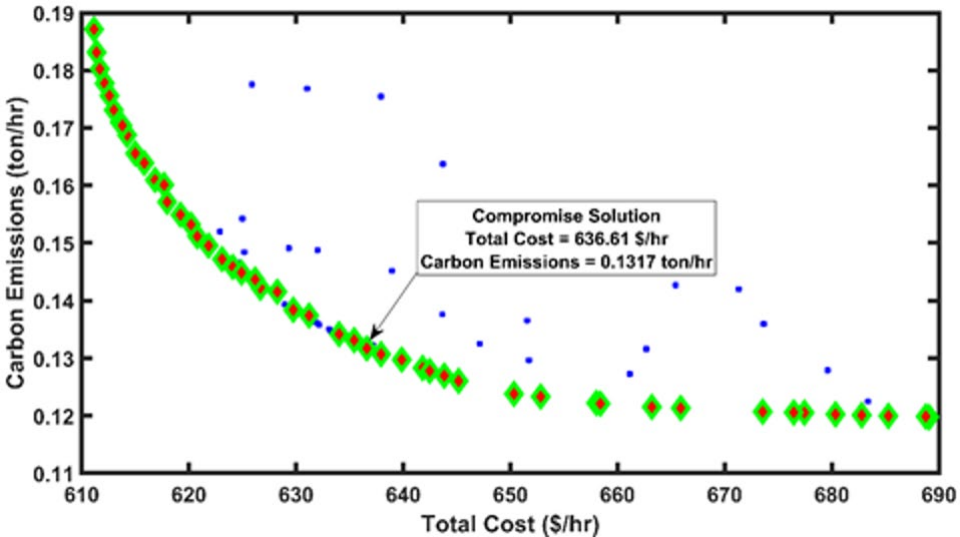
Pareto optimal solutions and the best obtained compromise solution at 35 °C.

**Fig. 24:**
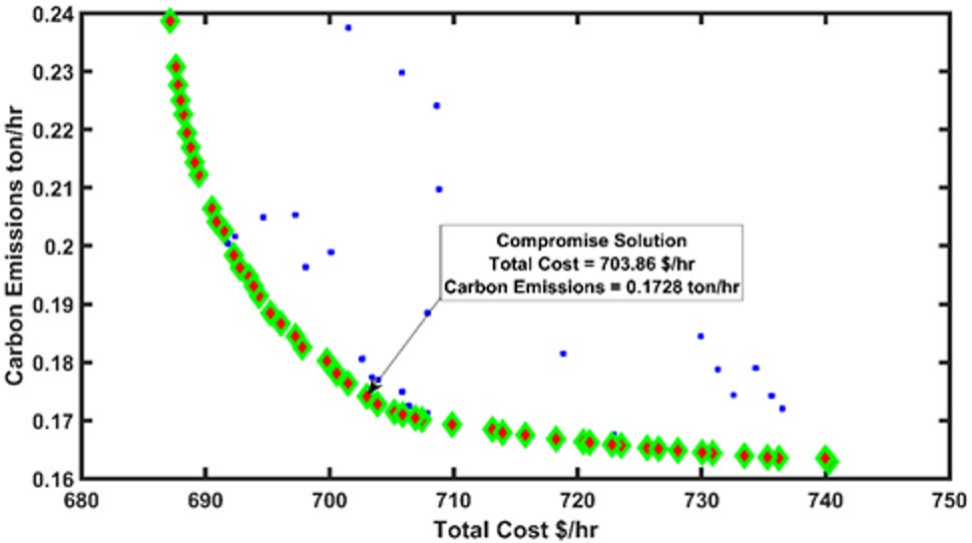
Pareto optimal solutions and the best obtained compromise solution at 40 °C.

In this part, the MO-SOPF problem with (total cost-carbon emission) minimization is presented at different temperatures at the beginning of the project. Figures 21, 22, 23 and 24 show the Pareto optimal solutions and the compromise solutions from 25 to 40 °C. Table 15 shows the compromise solution at different temperatures as well as the control variables.

**Table 15:** The compromise solution and control variables at different temperatures.

Parameter	$25^\circ C$	$30^\circ C$	$35^\circ C$	$40^\circ C$
${P}_{G1}$	78.659	79.91	94.71	134.04
${P}_{2-wind}$	77.256	77.504	67.116	40
${P}_{5-wind}$	49.255	48.345	42.45	25
${P}_{G8}$	32.03	31.063	34.153	34.88
${P}_{G11}$	22.684	24.124	23.6	29.86
${P}_{13-PV}$	27.75	26.74	26.11	25.98
Total cost	**603.386**	**609.342**	**636.61**	**703.86**
Carbon emission	**0.1218**	**0.1222**	**0.1317**	**0.1728**

Bold for the objective function in the optimization.

### Case XI: multi-period multi-objective SOPF considering ambient temperature and degradation effects

**Fig. 25:**
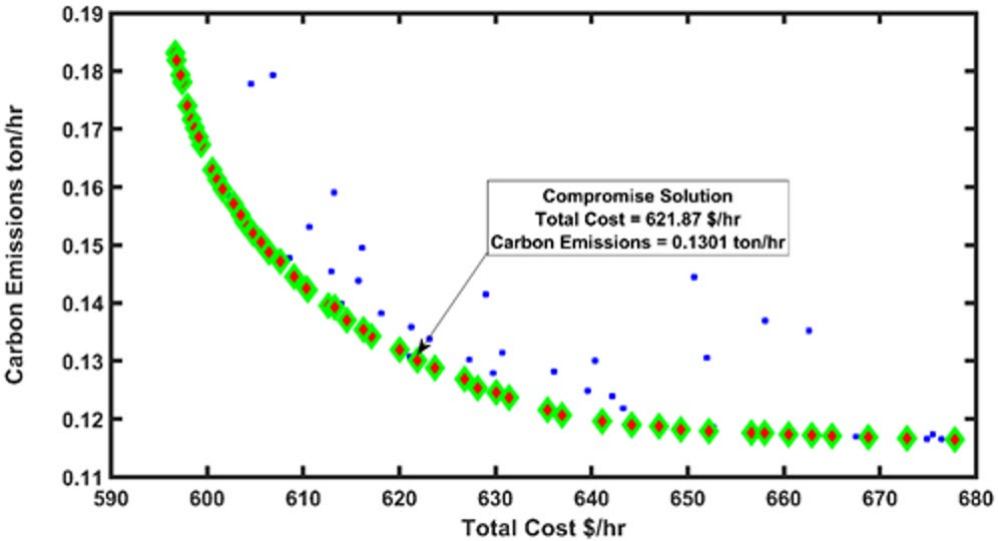
Pareto optimal solutions and the best obtained compromise solution at 25 °C (Year 15).

**Fig. 26:**
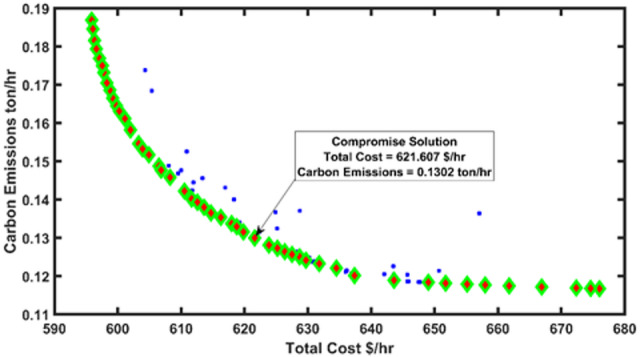
Pareto optimal solutions and the best obtained compromise solution at 30 °C (Year 15).

**Fig. 27:**
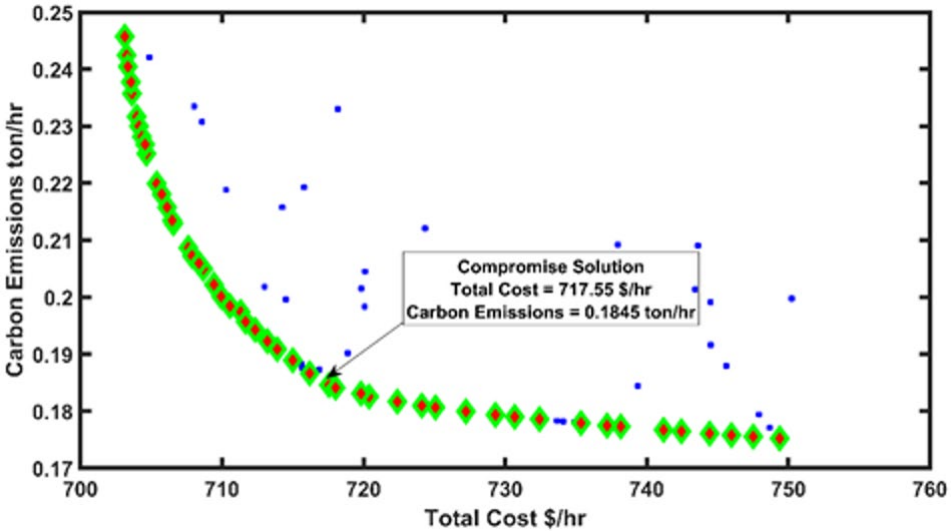
Pareto optimal solutions and the best obtained compromise solution at 40 °C (Year 15).

**Fig. 28:**
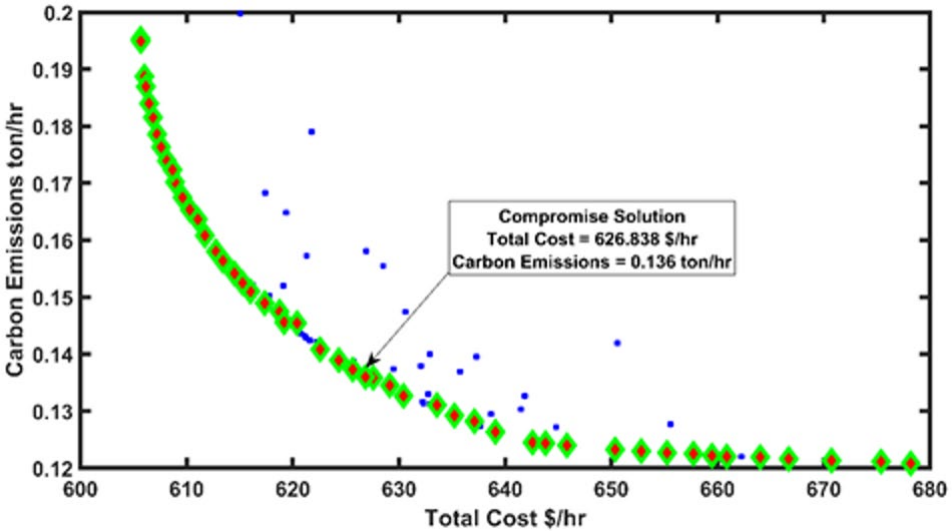
Pareto optimal solutions and the best obtained compromise solution at 25 °C (Year 25).

**Fig. 29:**
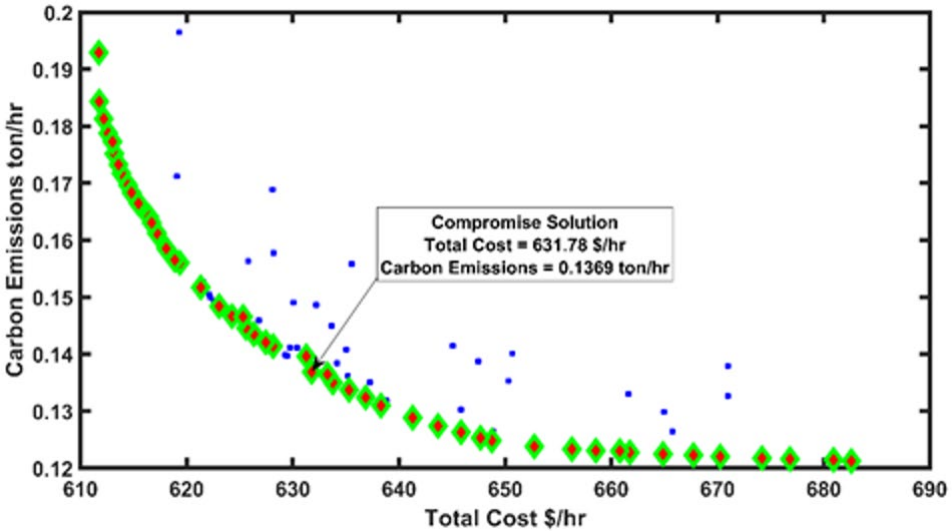
Pareto optimal solutions and the best obtained compromise solution at 30 °C (Year 25).

**Fig. 30:**
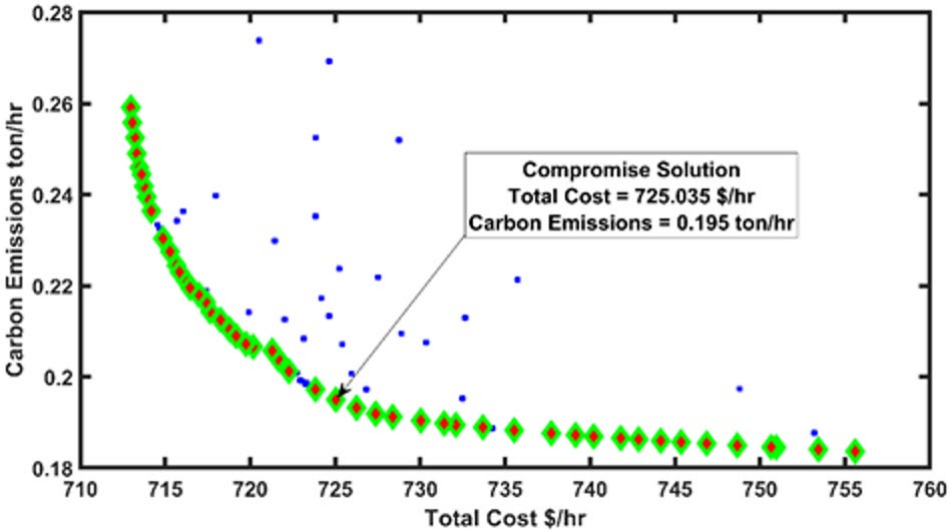
Pareto optimal solutions and the best obtained compromise solution at 40 °C (Year 25).

In this part, the multi-period MO-SOPF problem with (total cost-carbon emission) minimization is presented at 25 °C, 30 °C and 40 °C to show the impact of temperature rise. Moreover, the degradation effect is considered where the multi-period MO-SOPF problem is solved at 15th and 25th year respectively. Figures 25, 26, 27, 28, 29 and 30 show the Pareto optimal solutions and the best obtained compromise solutions at 25 °C, 30 °C and 40 °C respectively. Tables 16 and 17 show the compromise solution at different temperatures as well as the control variables at 15th and 25th year respectively.

**Table 16:** The compromise solution and control variables at different temperatures (Year 15).

Parameter	$25^\circ C$	$30^\circ C$	$40^\circ C$
${P}_{G1}$	91.376	91.427	141.735
${P}_{2-wind}$	72.33	72.016	36.55
${P}_{5-wind}$	45.65	45.525	22.84
${P}_{G8}$	32.33	32.8	35
${P}_{G11}$	21.3	21.424	30
${P}_{13-PV}$	25.335	24.6	24
Total cost	**621.607**	**621.876**	**717.55**
Carbon emission	**0.13**	**0.1301**	**0.185**

Bold for the objective function in the optimization.

**Table 17:** The compromise solution and control variables at different temperatures (Year 25).

Parameter	$25^\circ C$	$30^\circ C$	$40^\circ C$
${P}_{G1}$	99.372	100.233	147.86
${P}_{2-wind}$	68.76	67.5	34.314
${P}_{5-wind}$	42.93	42.723	21.506
${P}_{G8}$	31.735	33.28	34.98
${P}_{G11}$	22.23	21.33	29.47
${P}_{13-PV}$	23.317	23.29	22.44
Total cost	**626.84**	**631.8**	**725.034**
Carbon emission	**0.136**	**0.137**	**0.195**

Bold for the objective function in the optimization.

## Conclusion

This study investigates the influence of rising ambient temperatures on power system operation, emphasizing cost and emissions objectives. Higher temperatures reduce the output of wind and solar PV units, thereby increasing reliance on thermal generators. As a result, fuel usage and carbon emissions rise, reducing the economic and environmental benefits of renewable energy. The study demonstrates that the Mayfly managed to estimate Weibull and Lognormal PDF parameters for wind speed and solar irradiance modeling managing to minimize the RMSE for better modeling. Moreover, it excels at solving conventional OPF problem achieving a 0.6% and 0.5% reduction in fuel cost and carbon emissions compared to various algorithms. In addition, the MA method is utilized to solve single and multi-objective SOPF problem on a modified IEEE-30 bus system with two wind farms and one solar PV farm. This work employs a renewable energy model that incorporates the influence of ambient temperature on wind turbine and solar PV units performance. Moreover, a novel contribution to the SOPF objective function through considering Carbon credit concept. The results show that when minimizing overall cost, considering ambient temperature de-rating leads to increases of 21% in total cost and 45.67% in carbon emissions compared to ignoring temperature effects. When minimizing carbon emissions, these increases reach 9.67%and 45.7% respectively. Moreover, Multi-period single objective SOPF problem is solved at different years of lifetime and at different ambient temperature. The results demonstrate that, after 25 years of operation, and at 40 °C ambient temperature, when minimizing overall cost, the total cost and carbon emissions increased by 24.96% and 51.8% respectively compared to the case when the degradation of renewable energy is not considered. Multi-objective SOPF problem is solved, the results show the impact of ambient temperature on the control variables and compromise solution, when the temperature reaches 40 °C, the compromise solution shows a 16.65% increase in total cost and a 41.87% rise in carbon emissions. In addition, Multi-period Multi-objective MPSOPF problem is solved considering the degradation effect. The results show the impact on both control variables and compromise solution, by the 25th year of operation, when the temperature reaches 40 °C, the total cost increases by 20.16% and the carbon emissions increases by 60.1% after 25 years compared to the case when the temperature rise and degradation effect are not taken into account. The proposed MA method has superior performance in solving OPF problem. Furthermore, this study illustrates the significant influence of climate change in addressing the SOPF problem and highlights the necessity of system operators take climate change and renewable energy degradation into account when solving SOPF issues. Where, the impact of climate-related renewable performance reduction can be mitigated through adaptive operational strategies, such as improved dispatch scheduling, energy storage integration, demand response programs, grid reinforcement, and repowering renewable energy projects. These measures can enhance system flexibility, reduce renewable curtailment, and limit the need for additional thermal generation during stressed operating conditions. Therefore, future work will extend the proposed framework to include storage systems, demand-side flexibility, and grid modernization options to provide a more comprehensive assessment of climate-resilient power system operation.

## Data Availability

The datasets used and/or analyzed during the current study are available from the corresponding author on reasonable request.
